# Supplementary material for: Regulation of CD19 CAR-T cell activation based on an engineered downstream transcription factor
Source: Mol Ther Oncolytics. 2023 Apr 26;29:77–90. doi: 10.1016/j.omto.2023.04.005 (PMC10200817; doi:10.1016/j.omto.2023.04.005)
Supplement: Document S1. Figures S1–S5, Tables S1, and S2 [file mmc1.pdf]

OMTO, Volume *29*

Supplemental information

Regulation of CD19 CAR-T cell activation based on an engineered downstream transcription factor

Duško Lainšček, Anja Golob-Urbanc, Veronika Mikolič, Jelica Pantović-Žalig, Špela Malenšek, and Roman Jerala

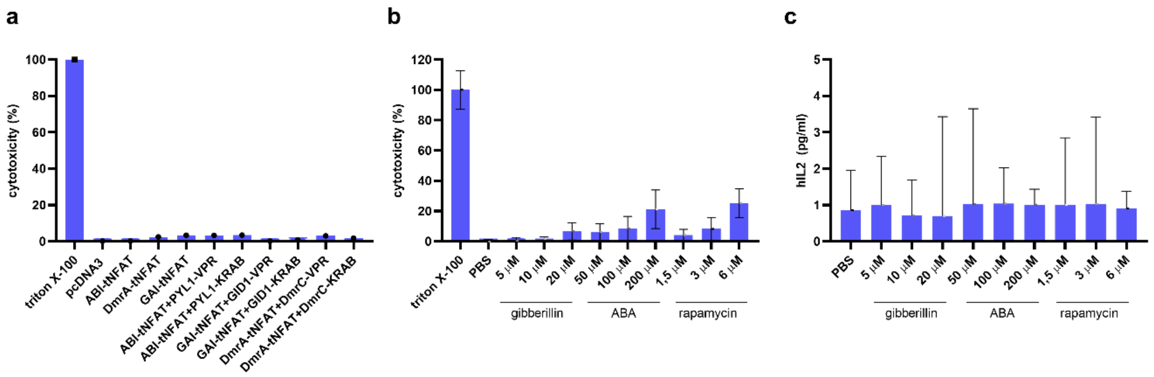

Figure S1: tNFAT transcription factors influence cell viability and activation. Jurkat cells ($3x10^7$ cells/ml) were Neon electroporated with listed plasmid DNA for expression of tNFAT transcription factors. 48 hours later LDH release was determined in cell media and % of cytotoxicity was calculated (a). Jurkat cells ($1x10^6$ cells/ml) were treated with various concentrations of heterodimerization inductors and 48 hours later cytotoxicity was determined based on LDH release (b) and IL2 secretion was quantified using ELISA (c). Data present three individual separate experiments (n=3).

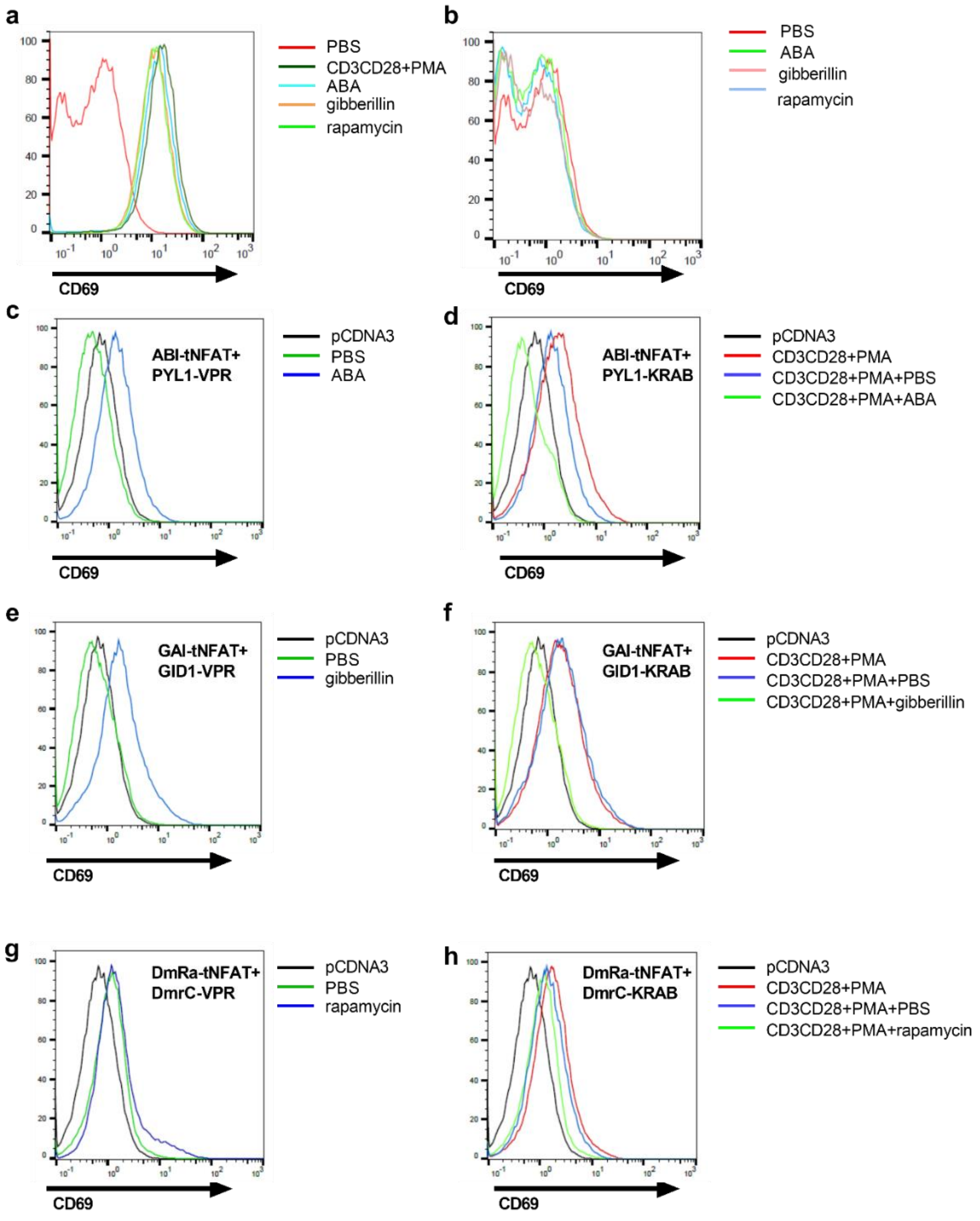

Figure S2: Regulation of T cell activation based on tNFAT transcription factors. Jurkat cells ($3x10^7$ cells/ml) were Neon electroporated with depicted plasmid DNA for expression of tNFAT transcription factors and stained against CD69 to determine cell activation. Jurkat cells were electroporated with empty pcDNA3 plasmid and 24 hours later treated with CD3CD28 Dynabeads (beads:cells=1:1)+PMA (50ng/ml). The next day cells were stimulated with HD inductors (ABA-100 μM; Gibberillin-10 μM or rapamycin- 3 μM). 24 hours later CD69 cell surface expression was determined (a). pcDNA3 electroporated Jurkat cells were stimulated with HD inductors and CD69 was measured (b). tNFAT transcription factors were electroporated into Jurkat cells and cell activation was determined 48 hours later after the

induction of heterodimerization with the addition of ABA (100 μM) (c-d), gibberellin (10 μM) (e-f) or rapamycin (3 μM) (g-h).

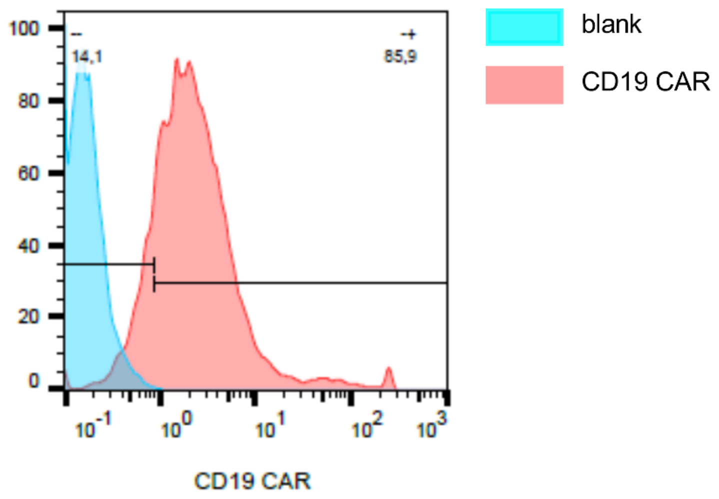

Figure S3: T cell transduction efficiency. To obtain CD19 CAR-T cells, T cells were transduced using retronectin-mediated retroviruses (CD19 CAR) or empty vector (blank). 5 days after the transduction, T cells were stained against the myc tag, expressed on the N-terminus of the CD19 CAR construct, and subjected to flow cytometry.

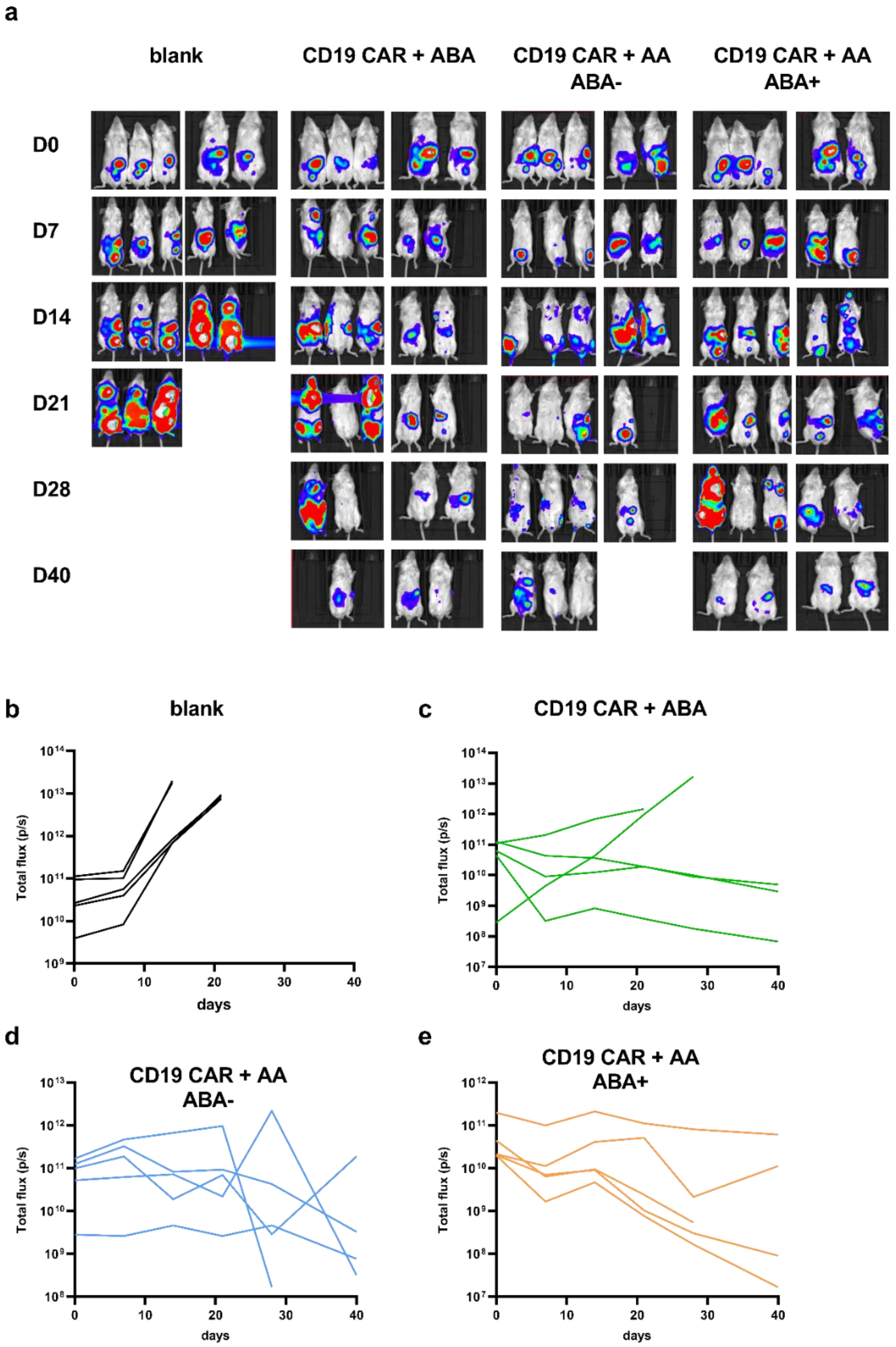

Figure S4: CD19 CAR T cell therapeutic efficiency *in vivo*. A hCD19+ BCWM-fLUC xenograft mouse cancer model was developed (10^6 cells/mouse). CD19 CAR T cells or ABA AA CD19 CAR T cells ($5x10^6$ cells/mouse) were administered. Daily ABA (100 μM) was injected into mice. Mice were subjected to BLI imaging weekly (a). Total flux measurement for cancer cell proliferation in vivo. Each line presents a single animal (b-e).

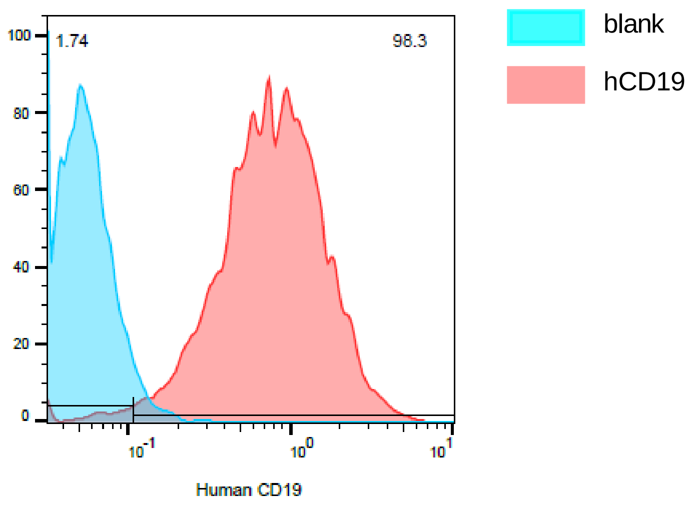

Figure S5: hCD19 MDA-MB-BR-231-fLUC cell generation. To obtain hCD19 MDA-MB-BR-231-fLUC cells, cells were transduced using lentiviruses (hCD19) or empty vector (blank). Three weeks after the puromycin selection, cells were subjected to flow cytometry to determine stable hCD19 integration.

Table S1: Statistical analysis for Total flux analysis (related to Fig 4h)

Tukey's multiple comparisons tests	Mean Diff.	Significant	Summary	P Value
blank vs. CD19 CAR+ ABA	3301934969444	No	ns	0,1066
blank vs. CD19 CAR+ AA (ABA-)	3923746600000	Yes	*	0,0441
blank vs. CD19 CAR+ AA (ABA+)	4095742344444	Yes	*	0,0341
CD19 CAR+ ABA vs. CD19 CAR+ AA (ABA-)	621811630556	No	ns	0,9552
CD19 CAR+ ABA vs. CD19 CAR+ AA (ABA+)	793807375000	No	ns	0,9129
CD19 CAR+ AA (ABA-) vs. CD19 CAR+ AA (ABA+)	171995744444	No	ns	0,9989

Ordinary one-way ANOVA was carried out with Tukey's multiple comparison test.

Table S2: Statistical analysis for Survival analysis (related to Fig 4i)

Log-rank (Mantel-Cox) test	Significant	Summary	P Value
blank vs. CD19 CAR+ ABA	Yes	**	0,0067
blank vs. CD19 CAR+ AA (ABA-)	Yes	*	0,0323
blank vs. CD19 CAR+ AA (ABA+)	Yes	**	0,0029
CD19 CAR+ ABA vs. CD19 CAR+ AA (ABA-)	No	ns	0,9065
CD19 CAR+ ABA vs. CD19 CAR+ AA (ABA+)	No	ns	0,5203
CD19 CAR+ AA (ABA-) vs. CD19 CAR+ AA (ABA+)	No	ns	0,5203

A comparison of survival curves was performed with Long-rank (Mantel-Cox) test.
