# Supplementary material for: Regulation of CD19 CAR-T cell activation based on an engineered downstream transcription factor
Source: Mol Ther Oncolytics. 2023 Apr 26;29:77–90. doi: 10.1016/j.omto.2023.04.005 (PMC10200817; doi:10.1016/j.omto.2023.04.005)
Supplement: Document S2. Article plus supplemental information [file mmc2.pdf]

Molecular Therapy
Oncolytics
Original Article

Regulation of CD19 CAR-T cell activation based on an engineered downstream transcription factor

Duško Lainšček,[1,2,5] Anja Golob-Urbanc,[1,5] Veronika Mikolič,[3,4] Jelica Pantović-Žalig,[1,4] Špela Malenšek,[1,4] and Roman Jerala[1,2]

[1]Department of Synthetic Biology and Immunology, National Institute of Chemistry, Hajdrihova 19, Ljubljana 1000, Slovenia; [2]EN-FIST Centre of Excellence, Trg Osvobodilne fronte 13, Ljubljana 1000, Slovenia; [3]Department of Hematology, Division of Internal Medicine, University Medical Center Ljubljana, Zaloška 7, Ljubljana 1000, Slovenia; [4]Graduate School of Biomedicine, University of Ljubljana, Ljubljana 1000, Slovenia

CAR-T cells present a highly effective therapeutic option for several malignant diseases, based on their ability to recognize the selected tumor surface marker in an MHC-independent manner. This triggers cell activation and cytokine production, resulting in the killing of the cancerous cell presenting markers recognized by the chimeric antigen receptor. CAR-T cells are highly potent serial killers that may cause serious side effects, so their activity needs to be carefully controlled. Here we designed a system to control the proliferation and activation state of CARs based on downstream NFAT transcription factors, whose activity can be regulated via chemically induced heterodimerization systems. Chemical regulators were used to either transiently trigger engineered T cell proliferation or suppress CAR-mediated activation when desired or to enhance activation of CAR-T cells upon engagement of cancer cells, shown also *in vivo*. Additionally, an efficient sensor to monitor activated CD19 CAR-T cells *in vivo* was introduced. This implementation in CAR-T cell regulation offers an efficient way for on-demand external control of CAR-T cell activity to improve their safety.

INTRODUCTION

Conventional cancer therapy is based on surgical intervention by resecting tumors in combination with radiation or chemotherapy to target rapidly progressive proliferating tumor cells. Even in this case patient's immune system plays a major role in cancer elimination. An important advance in cancer immunotherapy occurred upon the introduction of tumor-specific T cell receptor (TCR) engineered T cells.[1] This therapy had, however, some limitations caused by human leukocyte antigen (HLA) restrictions, restricting application to modification of patient's cells. Another breakthrough in cancer immunotherapy occurred when chimeric antigen receptor T (CAR-T) cells were introduced as an MHC-independent adaptive T cell therapy,[2,3] making those cell products easier to generate by using principles of synthetic biology.[4] The success of CAR-T cell therapy against CD19 malignancies led to the approval of this drug by the US Food and Drug Administration (FDA).[5] However, this highly efficient immunotherapeutic approach carries some risks that have to be addressed. One of the largest life-threatening CAR-T cell-associated adverse effects is a cytokine release syndrome and other excessive CAR-T cell activation-induced toxicities.[6] Other drawbacks of CAR-T cell therapy are limited efficacy against solid tumors with connecting immunosuppressive tumor microenvironment, inhibition and resistance in B cell malignancies, limited persistence, cell trafficking, and poor tumor infiltration.[6–8] To overcome some of those issues, a greater number of immunotherapeutic cells can be infused[9]; however, externally regulated proliferation of therapeutic T cells would be more desirable. For wider application and application at earlier disease stages, the safety and control of CAR-T cell-based therapy needs to be improved. Augmented CAR-T cell proliferation and activation has been introduced based on the addition of T cell growth factors,[9] which may, however, not act specifically just on CAR-T cells. For enhanced killing efficiency armored CAR or TRUCK cells were developed[10] implementing transgene expression cassettes, which resulted in high cell-mediated mediated cytotoxicity.[11–14] For the downregulation of CAR-T cell function, negative regulators have been used, based on antibody-based, inhibitors, kill switches, or inhibitory domains, such as programmed cell death 1 (PD-1) or cytotoxic T lymphocyte-associated (CTLA)-4 and so on, that have been incorporated into CAR constructs.[15–18] Additional improvements were made by designing synthetic receptors comprising small molecule-inducible systems, which regulated expansion and survival of CAR-T cells.[19–23] By implementing a synthetic biology approach into the design of synthetic CAR constructs, genetic circuits[7,24] were introduced to control therapeutic properties of CAR-T cells, where external control over infused therapeutic cells can be achieved.

Received 5 January 2022; accepted 24 April 2023;
https://doi.org/10.1016/j.omto.2023.04.005.

[5]These authors contributed equally

Correspondence: Duško Lainšček, Department of Synthetic Biology and Immunology, National Institute of Chemistry, Hajdrihova 19, Ljubljana 1000, Slovenia.
E-mail: dusko.lainscek@ki.si
Correspondence: Roman Jerala, Department of Synthetic Biology and Immunology, National Institute of Chemistry, Hajdrihova 19, Ljubljana 1000, Slovenia.
E-mail: roman.jerala@ki.si

This is an open access article under the CC BY license (http://creativecommons.org/licenses/by/4.0/).

Here, we developed a method for the external control over the activity of CD19 CAR-T cells based on engineered endogenous transcription factors (TFs) acting downstream of the CAR signaling pathway. A key TF of T cell signaling, nuclear factor of activated T cell 2 (NFAT2)[25] was selected, truncated and fused to binding domains of heterodimerization systems (HDs), whose activity can be regulated by the external addition of a small chemical compound. The corresponding protein binding partners of HDs were fused to the transcription activator or repressor domains to regulate the expression of NFAT-driven genes. We demonstrated control over the activity and functionality of T cells and CD19 CAR-T cells, which resulted in controlled killing of target cancer cells. Additionally, *in vivo* efficacy of the system was demonstrated in an animal xenograft cancer model. Furthermore, a CD19 CAR construct, co-expressing full-length NFAT exhibited augmented cancer immunotherapeutic properties. Finally, the NFAT-regulated reporter plasmid was also implemented as a sensor for the *in vivo* imaging of active CAR-T cells, thus providing a tool to monitor distribution and proliferation of activated CAR-T cells within an organism.

RESULTS

Protein-protein interaction domains have been already implemented in CAR-T cells to control their activity or provide target specificity. Different physicochemical inducers, such as light[26] or small molecules[27–33] (e.g., rapamycin, gibberellin,[20] A1120,[28] lenalidomide,[15] human retinol binding protein 4, grazoprevir, and danoprevir[34,35]) have been applied to control the functionality of CAR constructs via recruitment of signaling domains to the recognition domain. Instead of focusing on the modulation of a CAR, we decided to engineer a downstream signaling mediator to either reversibly trigger activation of engineered T cells or suppress their activation by the administration of a small molecule regulator. For this purpose, we used NFAT2 protein-based TFs. NFAT2 is one of the five members of NFAT family, of which NFAT1, 2, and 4 are key regulators of T cell function. NFATs contain a highly conserved DNA-binding domain, structurally related to the REL family TFs. REL-homology region confers a conserved DNA-binding specificity, whereas the second domain of NFAT is the NHR (NFAT-homology) domain that contains a potent transactivation domain. Upon TCR stimulation, a signaling cascade is activated, which results in a Ca^{2+} influx-dependent calcineurin-mediated dephosphorylation of serine residues of NFAT, exposing the nuclear localization signal, which results in the translocation of NFAT into the cell nucleus, where it promotes transcription of genes involved in T cell proliferation and activation, most prominently cytokines IL-2, tumor necrosis factor α, interferon (IFN) γ, IL-4, and several others.[25,36,37] Cytokine IL-2, which is secreted as a result of NFAT-mediated signaling pathway, acts as a strong inducer of T cell activation and proliferation.[38]

Here we engineered an endogenous TF NFAT2 (isoform C-α; 943 AA) so that it can target the same set of genes as the endogenous NFAT2, with the possibility of triggering CAR-T cell repression or activation by chemically inducible heterodimerization. The full-length NFAT2[25] was truncated by deletion of its transcriptional activation domain, resulting in a form $tNFAT2_{1-593}$ (hereafter referred to as tNFAT).[39] Next, tNFAT coding sequence was genetically fused at the N-terminus to domains of different HDs, resulting in a DmrA-tNFAT (part of the rapalog-inducible HD-DmrA and DmrC),[20] GID-tNFAT (part of the gibberellin inducible HD-GID and GAI1)[20,21] and ABI-tNFAT (part of abscisic acid-inducible HD-ABI and PYL1).[40] The interaction counterparts (DmrC, GAI1, and PYL1) were fused to a strong tripartite transcriptional activator domain VPR[41] (a NFAT activator) and/or strong repressor domain KRAB[42] (a NFAT repressor) (Figure 1A). To test the activity of designed TFs, we developed[39] a 3 × NFAT binding domain fLUC reporter plasmid with a minimal or constitutive promotor (Figure 1B) to monitor transcriptional regulation of fLUC. NFAT TFs were initially tested on HEK293 cells. The Ca^{2+} influx was provided by $CaCl_2$ buffer and Ca-ionophore stimulation. Upon gibberellin, abscisic acid (ABA) or rapamycin stimulation, we observed high upregulation of luciferase expression when cells were transfected with NFAT activator, tNFAT + VPR, genetically fused to a protein-protein interacting domain of HD (Figures 1C–1E). The statistically significant fLUC upregulation was achieved only in the presence of an appropriate inducer of a respective HD. An opposite action of engineered TF is the possibility to downregulate a gene of interest. This was analyzed by a reporter comprising a constitutive cytomegalovirus (CMV) promotor with adjacent NFAT binding sites. Decreased reporter activity in HD inducer-treated cells was observed for all three different NFAT-based designed TFs (Figures 1F–1H), demonstrating that designed NFAT TFs can trigger either positive or negative transcription regulation of NFAT-controlled genes.

Based on the results on HEK293 cells we aimed to regulate T cell function by NFAT-based TFs (Figure 2A). To determine T cell activation, Jurkat, a T cell line, was stimulated with CD3/CD28 antibodies in the presence of PMA. Upregulation of the activation marker CD69 and enhanced production and secretion of IL2 cytokine was observed, confirming successful endogenous TCR stimulation (Figure 2B; Figure S2A). The addition of HD inducer did not change CD69 expression, validating that CD69 upregulation indeed occurs because of TCR stimulation (Figure S1B). To achieve NFAT repressor-mediated IL2 downregulation, Jurkat cells were electroporated with NFAT repressors based on ABA, gibberellin, or rapamycin inducible HDs. Upon addition of the HD inducer to stimulated cells, strong attenuation of IL2 secretion was observed (Figure 2B). In contrast, when using NFAT activators we determined augmented IL2 production upon stimulation with ABA (Figure 2C), gibberellin (Figure 2D), or rapamycin (Figure 2E). Therefore, engineered NFAT TFs can be used as potent positive or negative transcription regulators of NFAT-driven genes, thus modifying T cell activation status as desired. This was also confirmed via fluorescence-activated cell source (FACS) analysis, where we found that CD69 expression correlated with the inducible assembly of NFAT activators or repressors (Figures S2C–S2H). Upon best activation via ABA of engineered TFs we observed not only cell activation increased, but also increased cell proliferation via *de novo* designed TFs (Figure 2F).

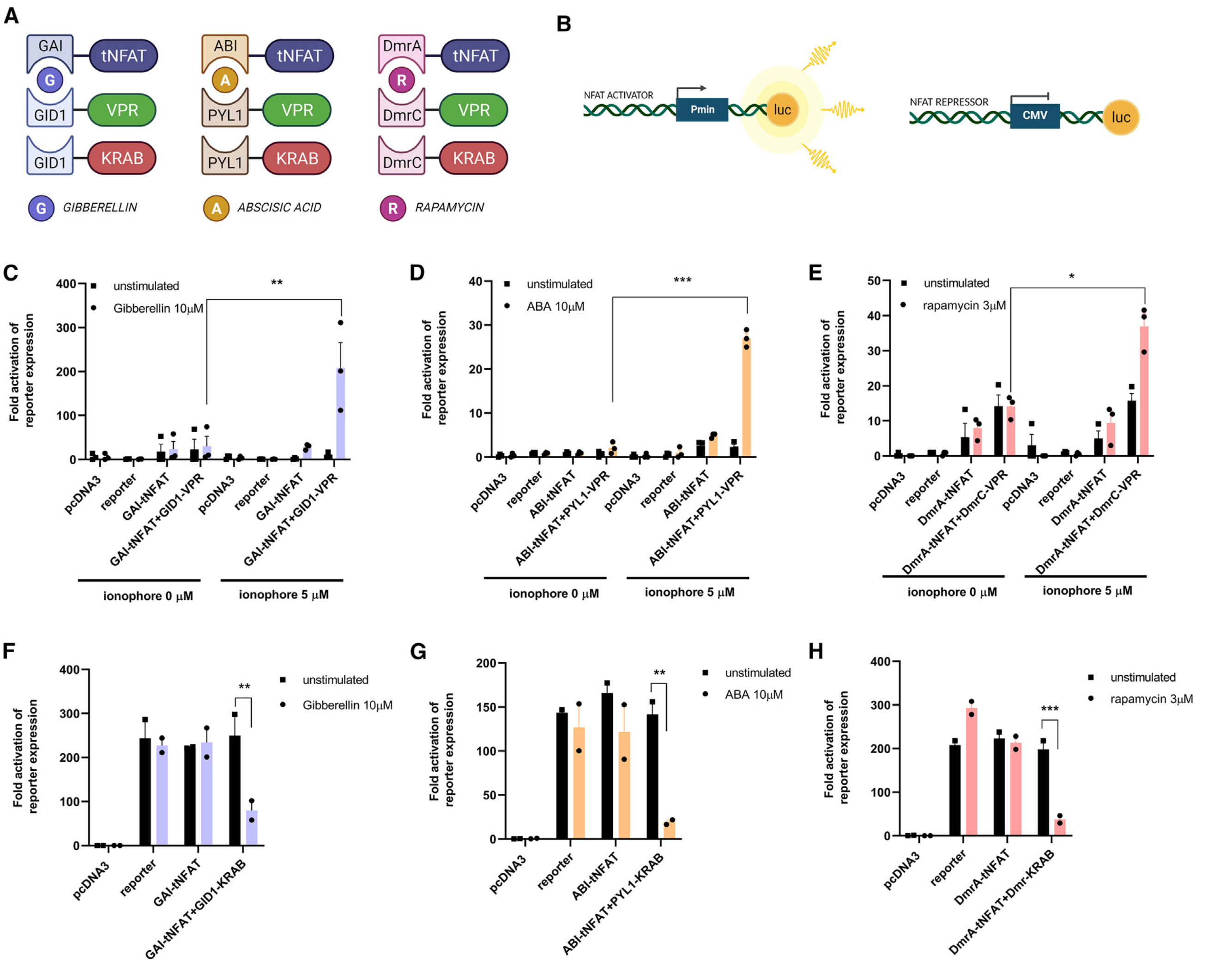

Figure 1. tNFAT transcription factors validation

Schematic representations of tNFAT TFs based on three different HD that influence gene transcription via activation (NFAT activator; tNFAT TF with VPR) or repressor domain (NFAT repressor; tNFAT TF with KRAB) (A). Experimental validation of tNFAT TF based on firefly luciferase (fLUC) expression regulated with minimal or constitutive promotor with three NFAT-binding sites upstream of the promotor (B). Influencing positive fLUC transcription using NFAT activators. HEK293 cells (2×10^4 cells/well) were transfected with NFAT activators and $_{3NFAT}$-P_{min} fLUC reporter plasmid and then in the presence of $CaCl_2$ with or without Ca-ionophore stimulated with gibberellin (10 µM) (C), ABA (100 µM) (D), or rapamycin (3 µM) (E). A relative luciferase assay was carried out 24 h later. Fold activation based on empty pcDNA3 vector transfection was calculated. fLUC transcription repression using NFAT repressors. HEK293 cells (2×10^4 cells/well) were transfected with NFAT repressors and $_{3NFAT}$-CMV fLUC reporter plasmid and then in the presence of $CaCl_2$ ± Ca-ionophore stimulated with gibberellin (10 µM) (F), ABA (100 µM), (G) or rapamycin (3 µM) (H). Relative luciferase was carried out 24 h later. Fold activation based on an empty pcDNA3 vector transfection was calculated. Data present three individual separate experiments (n = 3). $^*p < 0.05$, $^{**}p < 0.01$, $^{***}p < 0.0001$. All p values are from ordinary one-way ANOVA followed by Tukey's multiple comparison test.

Next, we tested if our engineered TFs carry any risk for cytotoxicity. We electroporated Jurkat cells with designated plasmid DNA (pDNA), coding for NFAT activators or repressors. By measuring lactate dehydrogenase (LDH) release we confirmed that engineered NFAT TF induced no cytotoxicity (Figure S1A). Empty vector electroporated Jurkat cells were stimulated with different concentrations of HD regulators to determine their possible influence on cytotoxicity and IL2 secretion. Only high doses of ABA and rapamycin resulted in an elevated release of LDH (Figure S1B), but we did not see any effect of inducers on IL2 secretion (Figure S1C), again stipulating that indeed only specific TCR stimulation or NFAT-mediated gene regulation leads to IL2 secretion and CD69 expression (Figure 2; Figure S2).

By demonstrating that T cell activity can be regulated via engineered tNFAT TFs, we wondered whether this could also be applied on CAR-T cells. Second-generation CD19 CAR construct, comprising CD19 scFv, 4-1BB costimulatory domain and CD3ζ[6] with ABA-inducible NFAT activators (AA) or repressors (AR),

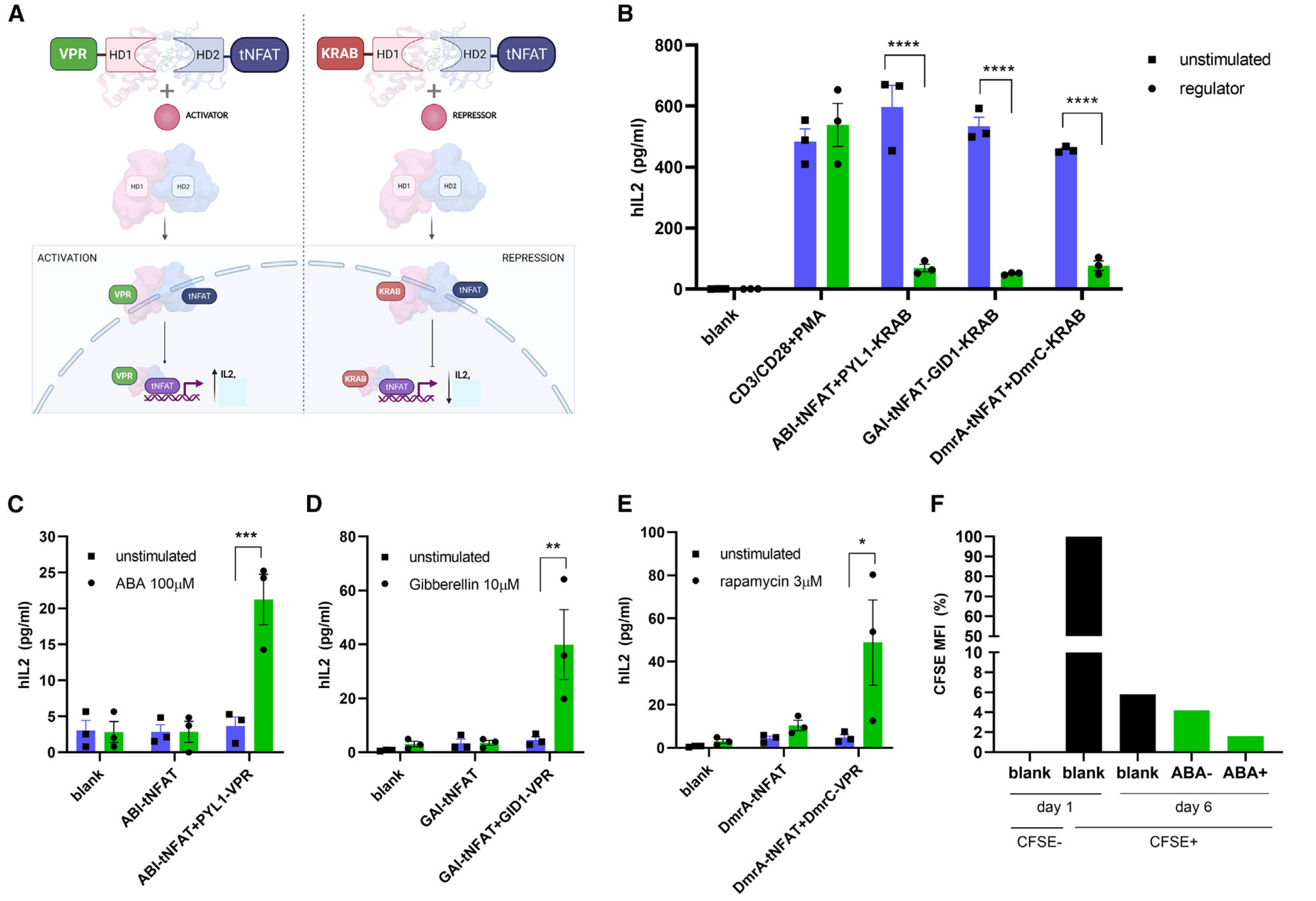

Figure 2. Engineered TFs regulate T cell activation status

Schematic presentation of tNFAT TF action. Upon the addition of regulator of heterodimerization, NFAT activators or repressor reconstitute. tNFAT TF nuclear import results in IL2 promotor binding and influencing *IL2* transcription (A). NFAT repressors suppress IL2 production in activated Jurkat cells. Jurkat cells (3×10^7 cells/mL) were pDNA electroporated and then activated with CD3CD28 Dynabeads (beads:cells = 1:1) + PMA (50 ng/mL). HD regulators (ABA-100 µM, gibberellin- 10 µM or rapamycin- 3 µM) were added 24 h later. IL2 was measured 48 h later (B). NFAT activators augment IL2 synthesis. NFAT activators were electroporated into Jurkat cells and then reconstitution of tNFAT TF was achieved via ABA (C), gibberellin (D), or rapamycin (E) addition. Proliferation status of Jurkat cells, electroporated with ABA-activator was observed at day 1 and day 6 (F). Data present three individual separate experiments (n = 3). *$p < 0.05$, **$p < 0.01$, ***$p < 0.0001$. All p values are from ordinary one-way ANOVA followed by Tukey's multiple comparisons test.

were delivered to Jurkat cells. We determined by ELISA that IL2 secretion upon co-culture of CD19 CAR-T cells with $CD19^+$ Raji target cells could be regulated by the addition of ABA. When stimulating co-cultured cells with ABA, we saw an increase in the IL2 synthesis when AA was used and IL2 decrease when AR was introduced into CD19 CAR-T cells (Figure 3A). A similar response was observed also for gibberellin-induced dimerization of designed tNFAT TFs (Figure 3B) and when rapamycin was applied as an inducer of *de novo* designed tNFAT TFs (Figure 3C). By adding heterodimerization inducers to CD19 CAR-T cells, co-cultured with target Raji cells, we observed that the addition of only rapamycin slightly diminishes IL2 secretion (Figure 3D), consistent with previous findings.[43]

As stated above, NFAT comprises its own activation domain[44] so we wondered if full-length NFAT2 ($_{fl}$NFAT) could be used as a costimulatory domain in CD19 CAR-T cell signaling. $_{fl}$NFAT was co-expressed with CD19 CAR construct in Jurkat cells, and target Raji cells were added. We observed that also $_{fl}$NFAT can act as an efficient co-stimulator of T cell activity as observed by an augmented IL2 production, which was not seen in cells that expressed only $_{fl}$NFAT (Figure 3E). This combination looks most promising for therapeutic application as it is composed of only human proteins and only requires a single polypeptide in addition to a CAR and provides a substantially potent response. However, it cannot be externally regulated. As the engineered NFAT TFs were designed to bind to the same genomic target as the endogenous NFAT,[45] we were intrigued if tNFAT without activation or repression domain could affect T cell activity. Jurkat cells were electroporated with CD19 CAR and variants of tNFAT with subsequent stimulation by Raji cells. We determined that T cell activity was attenuated for all three variants of tNFAT in a

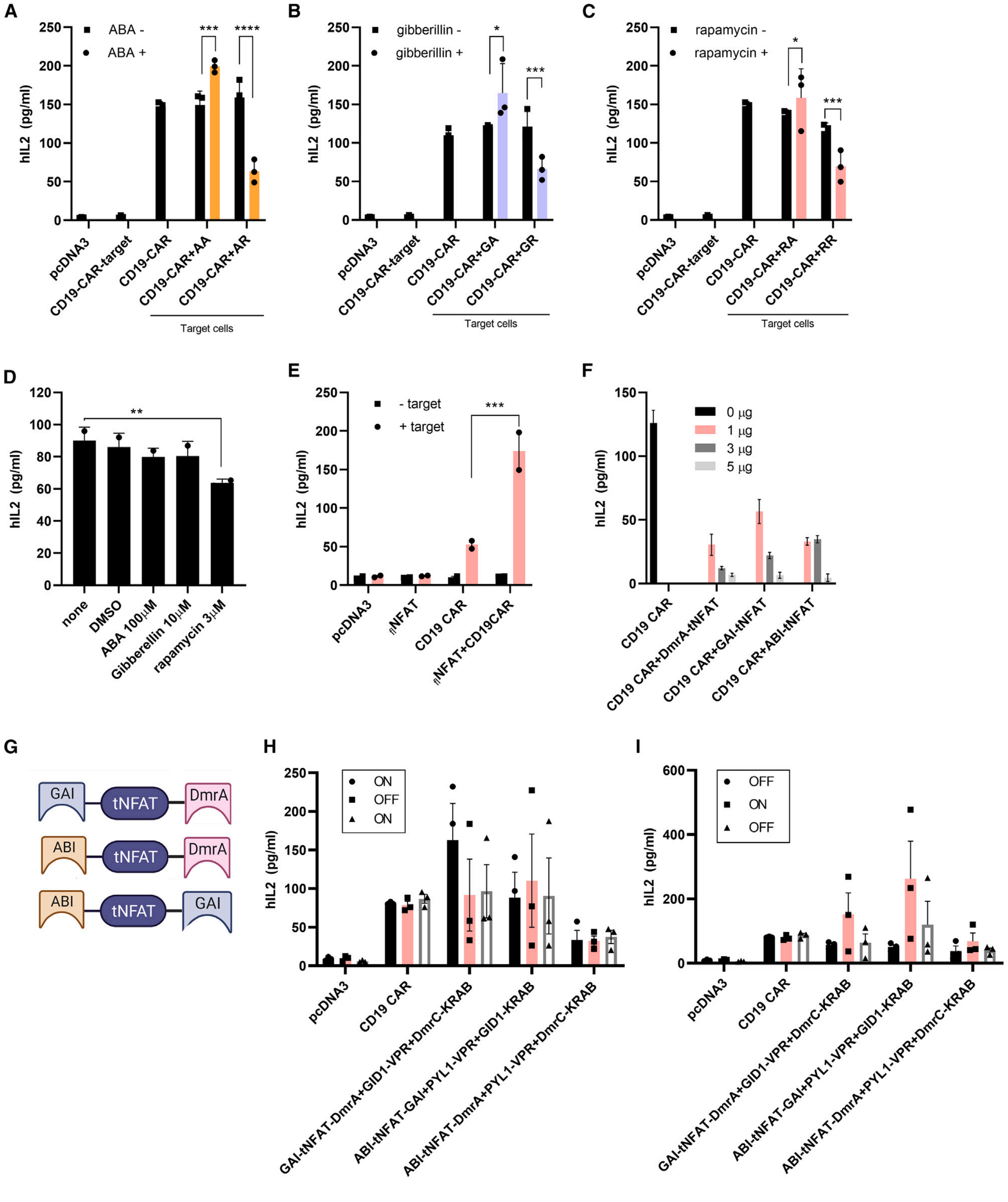

Figure 3. tNFAT TF mediated regulation of CD19 CAR-T cells

NFAT TFs influence CD19 CAR-T cell activation. Jurkat cells (3×10^7 cells/mL) were electroporated with pDNA, expressing CD19 CAR construct and tNFAT-based activators or repressors. The next day, cells were co-cultured with target CD19$^+$ Raji cells (effector:target = 10:1) and stimulated with chemical inducer of HD (ABA, 100 μM; gibberellin, 10 μM; or rapamycin, 3 μM). IL2 was measured 48 h later (A–C). The effect of the chemical inducer of HD activity on IL2 production. Jurkat cells (3×10^7 cells/mL)

(legend continued on next page)

concentration-dependent manner, most likely via competition of the designed tNFAT with an endogenous NFAT (Figure 3F), demonstrating that tNFAT can be also used as a negative regulator of CD19 CAR-T cell activity.

An efficient regulatory system should exhibit reversibility so that therapeutic activity can be turned on or off, so that the therapy could resume later. To provide a reversible CD19 CAR-T cell activity regulation system, we prepared a tripartite tNFAT TF, fused to two different HDs. tNFAT was, therefore, fused at the N- and C-terminus with GAI and DmrA domain, ABI and DmrA domain, or with the ABI and GAI domain of a certain system for induced heterodimerization (Figure 3G). To test the reversibility of the system, we electroporated Jurkat cells with CD19 CAR, tNFAT fused to GAI and DmrA and with gibberellin-inducible activator and rapamycin-inducible repressor domain. For the ON-OFF-ON switch experiment, cells were first stimulated with gibberellin to induce transcription and in the next stage with rapamycin to repress transcription. To turn the system ON again, cells were stimulated with gibberellin for the second time. The same ON-OFF-ON schedule was used for cells, bearing tripartite ABI-tNFAT-GAI TFs with co-expressed PYL1-VPR and GID1-KRAB or bearing ABI-tNFAT-DmrA with PYL1-VPR and DmrC-KRAB. To test the OFF-ON-OFF schedule, cells were electroporated with the same components as in the ON-OFF-ON schedule, but the stimulation protocol with chemical inducers was modified according to the desired outcome. Based on IL2 measurements, we concluded that the reversibility of the CAR-T cell response could be achieved, but we observed that rapamycin reversibility is harder to achieve in accordance to some published data[46] (Figures 3H and 3I).

As lymphoma and leukemia patients are treated with autologous CD19 CAR-T cells,[47] we set out to apply our regulatory system to primary human CD3$^+$ cells. Human T cells from healthy donors were retrovirally transduced with CD19 CAR, and transduction efficiency was determined by flow cytometry analysis with α-myc CD19 CAR staining (Figure S3). Based on the best results, obtained on Jurkat cells we selected ABA-inducible system to regulate CD19 CAR-T cell activity. By simultaneous transduction, different tNFAT TFs were introduced into CD19 CAR-T cells. After cell expansion, they were cultured with CD19$^+$ target fLUC-expressing BCWM cells, derived from a patient with Waldenström's macroglobulinemia, a B cell lymphoma.[48] Upon the addition of ABA to CD19 CAR-T cells, modified with ABA-induced tNFAT TFs, a regulated increase or decrease of IL2 and IFNγ secretion was observed, corresponding with the used NFAT activators or repressors (Figures 4A and 4B). Most important, also cancerous cell killing was regulated according to the tNFAT TFs used, demonstrating that not only cytokine production of therapeutic CD19 CAR-T cells could be modulated, but also their functionality (Figure 4C). It is well-known fact that different subsets of differentiated T cells exhibit various properties, for instance, effector T cells have an augmented killing ability, whereas a less differentiated T cell signature is associated with superior anti-cancer activities.[49] We, therefore, wondered if this augmented killing of CD19 CAR-T cells when ABA activators are present is caused by an altered phenotype of T cells. To test that, we rigorously stimulated CD19 CAR-T cells with ABA activators or solely CD19 CAR-T cells for 1 week. CD45RA and CD62L staining was carried out afterward that confirmed that ABA or the presence of ABA activators does not alter T cell phenotype compared with conventional CD19 CAR-T cells (Figure 4D), suggesting that ABA directly influences CD19 CAR-T function via NFAT TFs.

As some chemical inductors can have negative or even immunosuppressive effects on T cells, such as rapamycin,[32] we tested if the addition of ABA bears any adverse effects on CD19 CAR-T cells. We stimulated CD19 CAR with different concentrations of ABA in co-culture with target Raji-fLUC cells and afterward checked the functionality of the CD19 CAR-T cells. We did not observe any changes in cytokine production or in killing capacity compared with cells that were not stimulated with ABA (Figures 4E–4G). As we determined *in vitro* that ABA-mediated assembly of NFAT TF induces stronger activation of CD19 CAR-T cells, we set out to test this also *in vivo*; therefore, we established a BCWM-fLUC xenograft cancer model. Mice that exhibit progressive cancer growth were treated with CD19 CAR-T cells or CD19 CAR-T cells with ABA activator components. Because of the absence of negative side effects of ABA on conventional CD19 CAR-T cells, those animals also received ABA daily. By bioluminescence *in vivo* imaging, we discovered that in all groups CD19 CAR-T cells exhibited immunotherapeutic properties (Figure 4H; Figure S4), but when analyzing the total flux of BLI emitted from cancer cells, the statistical significance was confirmed only in ABA groups (Table S1). In addition, mice in the group that received CD19 CAR-T cells with ABA activators showed a lower final tumor burden and the survival rate was significantly higher (Figure 4I; Table S2).

were pDNA electroporated with CD19 CAR construct. Raji cells (effector:target = 10:1) and HD inducers (ABA,100 μM; gibberellin, 10 μM; or rapamycin, 3 μM) were added 24 h later. IL2 was measured 48 h later (D). The synergistic effect of full-length NFAT on T cell activation. Jurkat cells (3 × 10^7 cells/mL) were electroporated with pDNA, expressing CD19 CAR construct and full-length NFAT. Next day cells were co-cultured with target CD19$^+$ Raji cells (effector:target = 10:1). IL2 was measured 48 h later (E). tNFAT competes with endogenous NFAT and inhibits T cell activation. Jurkat cells (3 × 10^7 cells/mL) were electroporated with pDNA, expressing CD19 CAR construct and different amounts of variants of tNFAT. The next day, cells were co-cultured with target CD19$^+$ Raji cells (effector:target = 10:1). IL2 was measured 48 h later (F). Schematic representations of tripartite tNFAT TF based on two different protein components from two different HD that influence gene transcription (G). Reversibility of HD induced control over CD19 CAR-T cell activation. Jurkat cells (3 × 10^7 cells/mL) were electroporated with pDNA, expressing CD19 CAR construct and tripartite tNFAT-based activators or repressors. The next day, cells were co-cultured with target CD19$^+$ Raji cells (effector:target = 10:1) and stimulated with chemical inducer of HD (ABA-100 μM or gibberellin, 10 μM) for 24 h. The next day, cells were washed and rapamycin (3 μM) or gibberellin were added to switch down the system. Cells were again washed and stimulated with ABA or gibberellin 24 h later to show ON-OFF-ON of the system. Supernatant for IL2 measurement was taken every 24 h (H). OFF-ON-OFF of the system (I). Data present three individual separate experiments (n = 3). *$p < 0.05$, **$p < 0.01$, ***$p < 0.0001$. All p values are from ordinary one-way ANOVA followed by Tukey's multiple comparisons test.

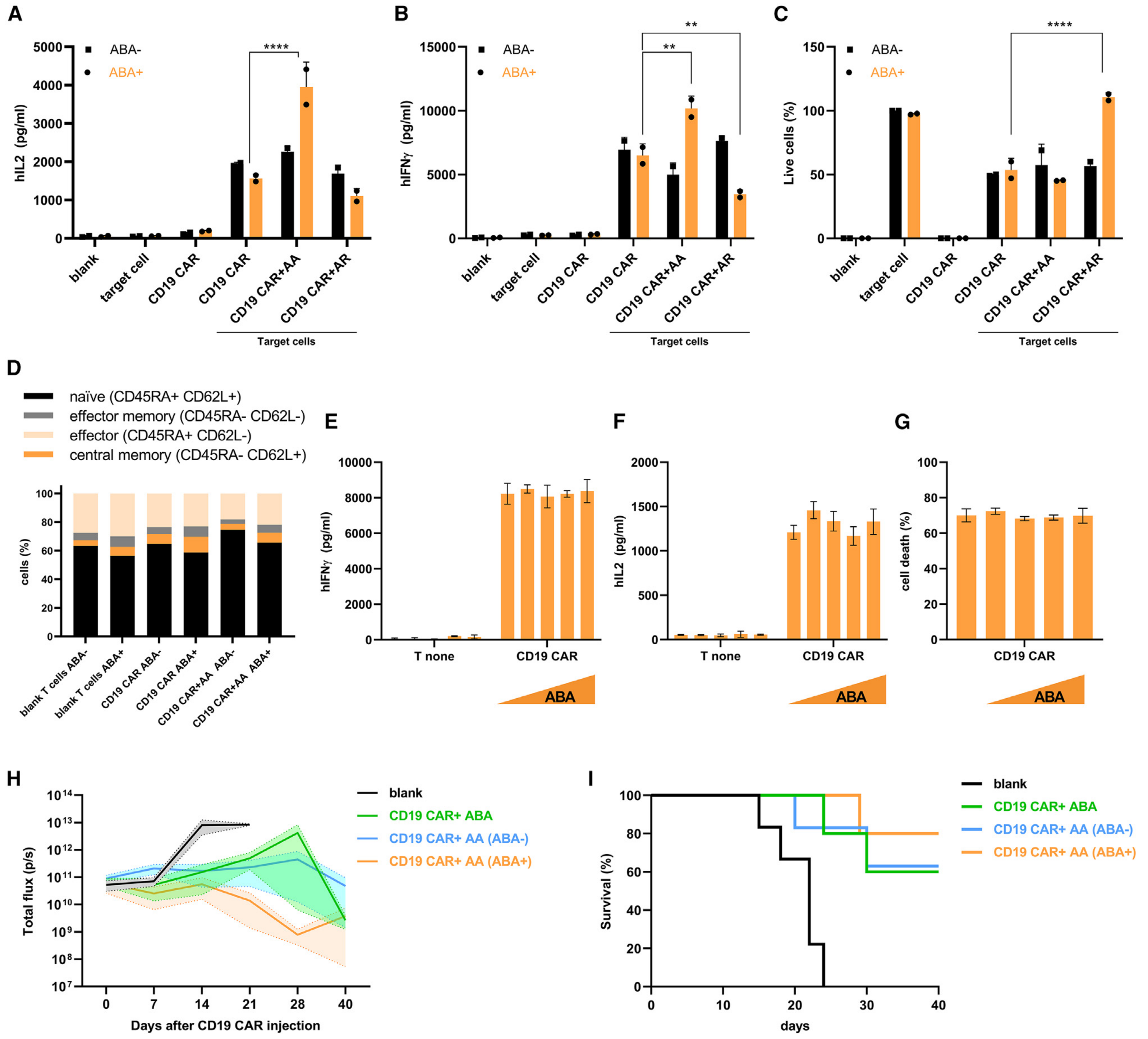

Figure 4. CD19 CAR-T cell function modulation via engineered tNFAT TF

NFAT TF regulate CD19 CAR-T cell activation and function. Human CD3$^+$ cells were virally transduced with CD19 CAR- and tNFAT-based activators or repressors. After appropriate time of cell expansion, CD19 CAR-T cells were co-cultured with target CD19$^+$ BCWM-fLUC (effector:target = 10:1) cells and stimulated with chemical inducer of HD (ABA, 100 μM). IL2 (A), IFNγ (B), and percent of live cells (C) was measured 48 h later. The percent of live cells was calculated based on drop of luminescence of target cells. Data present two individual separate experiments, where CD3 bulk cells originate from two different donors (n = 2). *p < 0.05, **p < 0.01, ***p < 0.0001, ****p < 0.00001. All p values are from ordinary one-way ANOVA followed by Tukey's multiple comparisons test. CD19 CAR and AA CAR cells phenotype determination. CD19 CAR or CD19 CAR AA cells were stimulated with 100 μM for 1 week. After that phenotype was determined via FACS (D). ABA addition does not influence functionality of CD19 CAR-T cells. CD19 CAR-T cells were stimulated with different concentrations of ABA (0, 10, 50, 100, and 150 μM) in presence of target BCWM-fLUC cells at E:T ratio = 5:1. IFNγ (E), IL2 (F) and killing (G) was determined. CD19 CAR AA exhibit higher *in vivo* efficiency. SCID mice received 10^6 Raji-fLUC cells. One week later, mice were administered with 5×10^6 CD19 CAR-T cells or CD19 CAR AA cells and daily dose of 100 μM ABA. BLI determination for tumor burden was carried out. Significance was determined by one-way ANOVA followed by Tukey's multiple comparisons test and are shown in Table S1 (H). Survival analysis for *in vivo* study. Significance was determined by long-rank test (Mantel-Cox) and are shown in Table S2 (I).

As we have shown that co-expression of $_{fl}$NFAT2 increases IL2 secretion in Jurkat cells (Figure 3E), we were wondering if this could correspond with augmented cancer killing in human CD19 CAR-T cells. We, therefore, transduced human T cells with viruses, expressing CD19 CAR constructed connected via t2a peptide to $_{fl}$NFAT2 (CD19 CAR_NFAT). Phenotype analysis via FACS revealed no

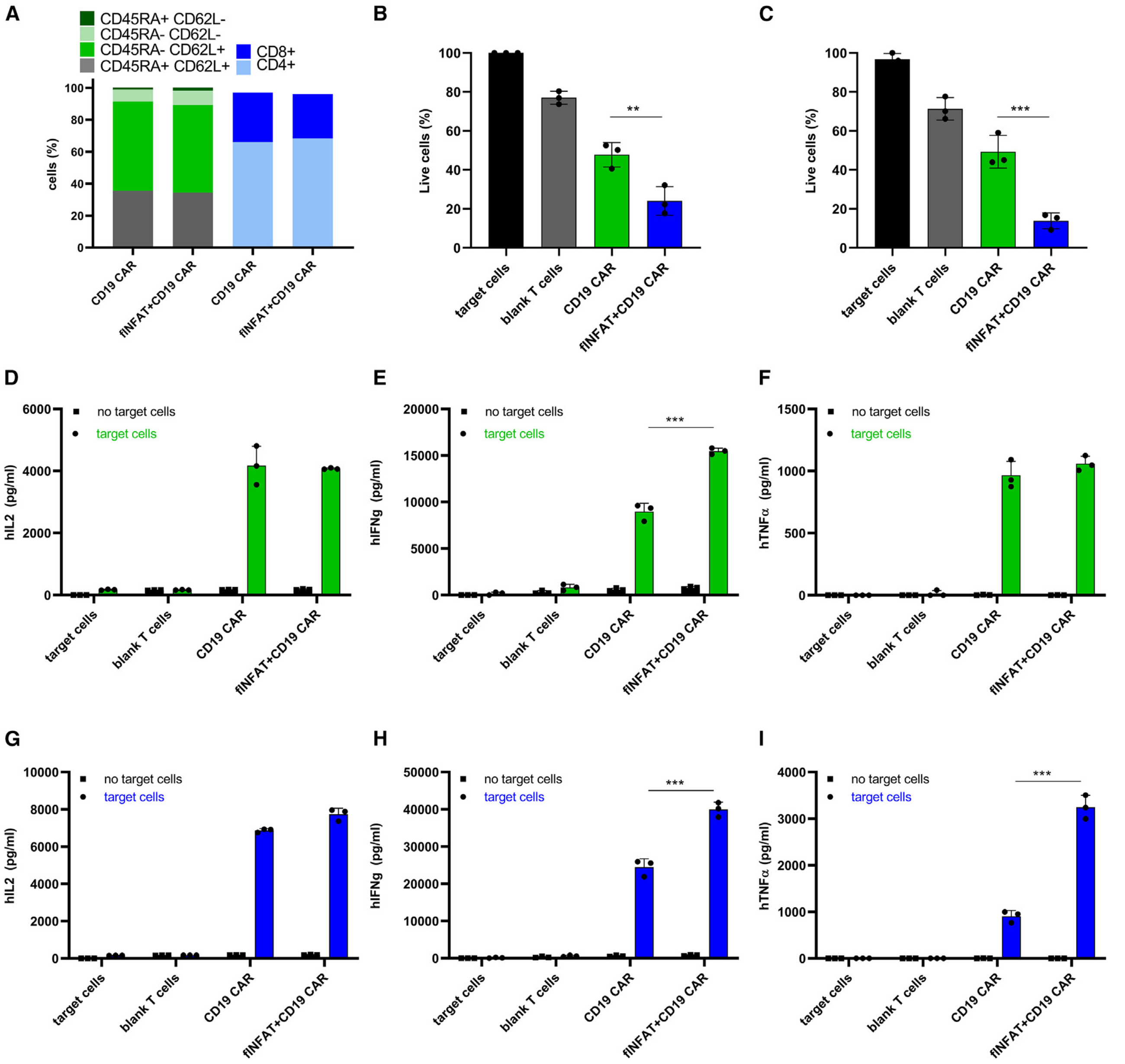

Figure 5. Increased cancer clearance of $_{fl}$NFAT2-expressing CD19 CAR-T cells

Co-expression of full-length human NFAT2 in CD19 CAR-T cells augments cancer immunotherapy. Human CD3^{+} cells were virally transduced with CD19 CAR or with CD19 CAR_t2a_$_{fl}$NFAT. After an appropriate time of cell expansion, the CD19 CAR-T cells phenotype was examined via FACS (A). CD19 CAR-T cells were co-cultured with CD19^{+} Raji-fLUC or hCD19-MDA-MB-231-BR cells (effector:target = 5:1). Killing efficiency was calculated based on exhibited BLI of Raji target cells (B) or for hCD19-MDA-MB-231-BR cells (C). IL2 (D), IFNγ (E), and tumor necrosis factor-α (F) were measured 48 h later when Raji cells were used as a target. IL2 (G), IFNγ (H), and tumor necrosis factor α (I) was measured 48 h later when hCD19-MDA-MB-231-BR cells were used as a target. ***$p < 0.0001$. All p values (n = 3) are from ordinary one-way ANOVA followed by Tukey's multiple comparisons test.

significant changes between conventional CD19 CAR and CD19 CAR_NFAT (Figure 5A). When co-cultured with Raji-fLUC target cells we saw an enhanced cancer killing of CD19 CAR_NFAT compared with CD19 CAR-T cells (Figure 5B). As solid cancer treatment in CAR therapy remains a challenge, we examined if augmented cancer killing could be also observed for the solid cancer cell line. We, therefore, prepared a human CD19-MDA-MB-231-BR-fLUC-expressing breast cancer model (Figure S5). We were pleased to observe enhanced cancer killing for the breast cancer cell line as well (Figure 5C). By ELISA measurements, we determined that cytokine

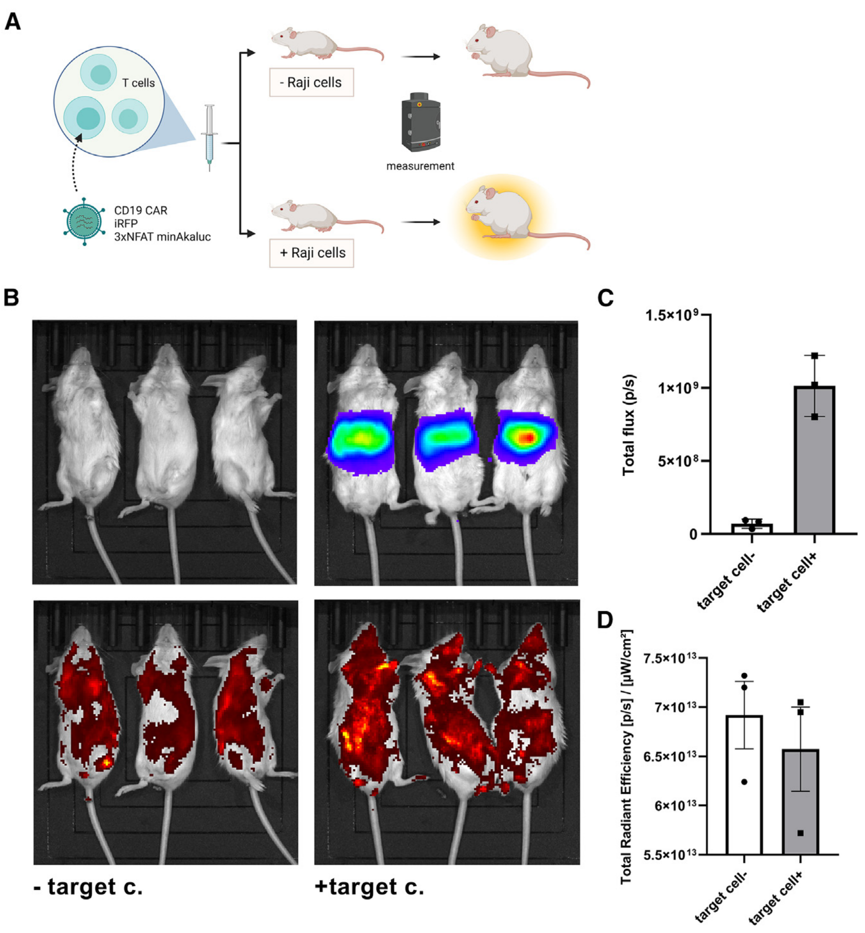

Figure 6. Visualization of CD19 CAR-Tcell activity based on Akaluc T cell sensor

Schematic representations of Akaluc based T cell sensor. Human CD3 cells were transduced with CD19 CAR, iRFP, and 3xNFAT minAkaluc. After 5 days of expansion, they were intraperitoneally injected into SCID mice. On the next day, target CD19$^+$ Raji cells were injected with PBS administered into the control group. BLI and fluorescence were determined 72 h later to monitor the presence and activation of CD19 CAR-T cells (A). IVIS imaging revealed increase of BLI signal upon Raji cells injection (B). Quantification of BLI values of activated CD19 CAR-T cells, bearing an Akaluc sensor (C). Fluorescence values, obtained *in vivo*, reflecting the presence of CD19 CAR-T cells (D).

secretion was increased in CD19 CAR-T cells, expressing $_{fl}$NFAT compared with conventional CD19 CAR, when Raji cells were used as a target (Figures 5D–5F) or hCD19-MDA-MB-231-BR were used (Figures 5G–5I).

One of the limiting factors of CAR-T cell solid tumor therapy is the poor distribution of T cells in the tumor.[50] CAR-T cell trafficking is typically monitored using fluorescently labeled cells.[51] This, however, does not reveal if the cells are activated or not; therefore, we aimed to use the 3xNFAT fLUC reporter plasmid developed here as a CD19 CAR-T cell activity sensor. Instead of fLUC, its derivative Akaluc was used, which has been proven as superior for the in *vivo* single cell analysis, trafficking, and tissue penetration.[52,53] CD19 CAR-T human cells, carrying CD19 CAR, 3xNFAT-Akaluc reporter, and iRFP were prepared. iRFP was included to visualize all CD19 CAR-T cells, while the expression of Akaluc should only correlate with CD19 CAR-T cell activation. CD19 CAR-T cells harboring a T cell sensor were injected into SCID mice. Raji cells were subsequently administered intraperitoneally to engage with CD19 CAR-T cells and activate Akaluc reporter, depending on NFAT activation. Three days later, animals were subjected to *in vivo* imaging, revealing that the bioluminescence signal was present only in the animals that were also injected with Raji cells (Figure 6A), suggesting that CD19 CAR-T cell activation is specifically detected via triggering a T cell sensor, whereas the iRFP signal was observed in all animals, demonstrating the presence of nonactivated CD19 CAR-T cells (Figures 6B–6D). Based on these results, we can conclude that the Akaluc-mediated T cell sensor can be used to study migration, trafficking, and location of activation of CAR-T cells, not only in hematological cancers, but it might also be extrapolated to study of cell trafficking within solid tumors.

DISCUSSION

Even though CAR-T cells are well-established as a therapeutic option in treating various forms of cancer, further improvements are needed to establish a controllable regulation over their activity to avoid side effects that can be life threatening.[29,47,50] Here, we implemented control of T cells based on a downstream endogenous regulator NFAT2.[54] NFAT2 was selected based on the fact that it is a key regulator of T cell activation and proliferation, promotes high cytotoxicity in tumor cytotoxic CD8$^+$ T cells, and has a great influence on cytokine signaling.[36,55] Unlike other NFATs, the isoform α enhances effector functions without promoting apoptosis of effector T cells.[56]

Engagement of NFAT enables several modalities to control the activity of CAR-T cells: (a) stimulation of CAR-T cells by target cancer cells can be substantially enhanced by co-expression with $_{fl}$NFAT, (b) CAR-T cells can be activated and proliferated by chemically regulated tNFAT activators, and (c) activity of CAR-T cells stimulated by target cancer cells can be suppressed by chemically regulated tNFAT repressors.

We discovered that all engineered variants of engineered tNFAT TFs could control IL2 production through NFAT activators and repressors, where ABA-inducible assembly showed best results in T cell line CARs, which was also confirmed in human CD19 CAR-T cells

and *in vivo*. By using the ABA-induced tNFAT TF regulating system in human CD19 CAR-T cells, we managed to control not only cytokine secretion, but also cancer cell killing. Most important, an efficient reversible negative regulation of IL2 and IFNγ secretion with subsequent impairment of cancer cell killing demonstrated that CAR-T cells can be turned off in case of excessive cytokine release, thereby avoiding potentially adverse effects, which needs to be tested in the future *in vivo* as well. By designing tripartite TFs, we additionally implemented an exogenous chemical control over CD19 CAR-T cell activity. This allows us to turn ON or OFF the therapeutic activity of CAR-T cells, an important feature of this system.[50] In the present study, different activation and deactivation schedules were tested, which could enable physicians to regulate CD19 CAR-T cell activity based on the patient's status. By using NFAT TFs that act upon two different inducers, we presented a conditional and orthogonal TF activity that can be extrapolated beyond NFAT and CAR-T cells. By switching to other regulatory proteins, we can influence not only cell differentiation state (e.g., changing to a more favorable phenotype of CAR-T cells, less exhausted T cells), but also cell fate alters promotor activities leading to controlled gene expression, altering the final therapeutic properties of advanced cell products. Because ABA-controlled activity of NFAT TFs exhibited the best effect, which was also demonstrated *in vivo*, where greater tumor clearance and survival were observed, we must comment on the potential drawbacks of ABA-inducible HD as it is of non-human origin. Li et al.[22] have shown that the PYL1 protein domain of the ABA CID has potential immunogenic properties, which could in principle be ameliorated by mutations based on the computational prediction of potential immunogenic epitopes to decrease the potential of MHCI presentation.[57] We believe that the potential immunogenicity of the system does not rule out the use of the presented approach; several other non-human protein components have been used in CAR-T control, for instance, viral-derived protease NS3a,[34] viral component HSV-TK (which are already in the clinical trials)[58] as well as bacteria-derived NAP protein,[59] among others. ABA was recognized also as a human hormone,[60] with low toxicity in mammals,[61] with an important positive role in glucose homeostasis[62] and important anti-inflammatory effects,[63] providing an added value to the use of ABA as a pharmacological control over therapeutic cells.

Although rapamycin-induced heterodimerizing tNFAT TFs showed promising results, the rapamycin could affect cell viability and functionality, since the increased LDH release and decreased IL2 secretion was observed when treating cells also by rapamycin only. It is also known that rapamycin exhibits immunosuppressive effects[32]; therefore, it may not be the best choice for CAR-T cell control. NFAT plays a complex role in T cells; as the constitutively active NFAT can drive T cell anergy and exhaustion by upregulating genes for the inhibitory surface molecule expression, such as PD-1, LAG3, CTLA-4, and so on.[64,65] Despite that, we did not observe impaired IL2 secretion, which is also one of the hallmarks of T cell exhaustion, when $_{fl}$NFAT was expressed together with CAR. This confirms that NFAT could be used as a costimulatory domain to some degree, whereas tNFAT can also act as a negative regulator based on the speculation that the engineered tNFAT could compete with an endogenous NFAT. $_{fl}$NFAT, co-expressed in human CD19 CAR-T cells exhibited augmented cancer immunotherapeutic properties not only for hematological cancer, but also as a platform for treating solid cancer.

Finally, we also constructed a genetic sensor to monitor or track activated CD19 CAR-T cells based on the integrated Akaluc-based T cell sensor, similar to that reported by others,[66,67] with an important improvement to track not only all T cells, but separately activated CAR-T cells as well. An Akaluc-based T cell sensor provides a tool to observe *in situ* activated CD19 CAR-T cells in real time for different types of tumors.

Altogether, we demonstrated the pharmacological regulation of engineered CAR-T cells that could provide increased safety due to the controllable activation and proliferation based on the inducible HDs targeting key TCR and CAR downstream regulators. As an alternative to the ABA-inducible HD system, NFAT TFs could be coupled to human-derived HDs to avoid possible immunogenicity issues, but it has to be stated that immunogenicity should not represent an obstacle in lympho-depleted CAR-T-treated patients; several potential immunogenic CAR-T constructs are already FDA approved and used in the clinic.[34] However additional study regarding the potential immunogenicity of this system needs to be and performed *in vivo*. Here we provided control of CD19 CAR-T cell activity based on an externally controlled downstream regulator to make CAR-T cell cancer immunotherapy more effective and safer.

MATERIALS AND METHODS

Plasmids

All plasmids used in this study were constructed using the Gibson assembly method. Human DNA fragment NFAT2 was synthesized by Genewiz. Truncated NFAT2 was cloned into pcDNA3 (Invitrogen) and connected at N-terminus via a 10 amino acid GS linker with domain ABI (PCR amplified from pSLQ2816 pPB [Addgene plasmid no. 84261]), GAI (PCR amplified from pSLQ2816 pPB [Addgene plasmid no. 84261]) or with DmrA (PCR amplified from pHet-NucI [iDimerize, Takara Bio USA]). The PYL1 and GID1 domains were PCR amplified from pSLQ2816 pPB (Addgene plasmid no. 84261), whereas DmrC was PCR amplified from pHet-I (iDimerize, Takara Bio USA). PYL1, GID1, or DmrC domains were connected at the C-terminus via a 10-amino acid GS linker with strong tripartite VPR activator (PCR amplified from pAG414GPDdCad9-VPR [Addgene plasmid no. 63801]) or with repressor KRAB domain (PCR amplified from pHR-SFFVdCas9-BFP-KRAB [Addgene plasmid 46911]). The reporter gene for firefly luciferase was PCR amplified from commercial plasmid pGL4.16 (Promega). The SV40 large T-antigen nuclear localization sequence hexahistidine tag, minimal promoter, and 3NFAT binding sites were introduced into the constructs with PCR. Renilla luciferase (phRL-TK; Promega) was used as a transfection control. For the T cell sensor, Akaluc was PCR amplified from pCDNA3-Venus Akaluc (Riken DNA Bank). The sequence for the CD19BBz construct was obtained from patent US20130287748 and cloned into pcDNA3 vector as a gBlock (IDT).

For retroviral transduction, the desired coding region was cloned into BamHI/EcoRI MCS of PMX-Puro (Cell Biolabs) retroviral vector. For lentiviral transduction, human CD19 (DNA fragment; Twist) was cloned into BamHI/EcoRI MCS of pLVX-Puro vector (Addgene, 141395). Additionally, pVSV-G (Addgene, 138479) and psPAX2 (Addgene, 12260) were used as a packing plasmids.

Cell cultures

The human embryonic kidney (HEK) 293, human Jurkat and Raji cells were purchased form American Type Culture Collection. HEK293 were cultured in DMEM (Invitrogen Life Technologies) supplemented with 10% (v/v) heat-inactivated FBS (Invitrogen Life Technologies), whereas Jurkat and Raji cells were grown in RPMI160 medium with 10% FBS. BCWM-fLUC cells were a kind gift from Steven P. Treon (DFCI) and grown in RPMI1640, supplemented with 10% fetal bovine serum (FBS). Human breast cancer cell line MDA-MB-BR-231-fLUC were a kind gift from Toni Petan and grown in DMEM medium, supplemented with 10% FBS.

For retrovirus production amphotrophic packaging cell line Gryphon Ampho (Allele Biotech) and for lentivirus production HEK293-T cells were used, which were grown in DMEM, supplemented with 10% FBS. Cells were cultured at 37°C in 5% CO_2.

T cells were obtained from healthy donors. Samples were obtained with informed consent, and according to the study protocol approved by the National Medical Ethics Committee (0120-21/2020/4). T cells were isolated from PBMCs by Ficoll Paque gradient centrifugation. Afterward Miltenyi PanT cell isolation kit was used to isolate $CD3^+$ cells according to the manufacturer's instructions. $CD3^+$ cells were maintained in RPMI medium, supplemented with 10% FBS, 25 μL/mL of ImmunoCult Human CD3/CD28 T cell activator (Stemcell) and 10 ng/mL of human IL2 (Preprotech) for five days before the viral transduction.

Luciferase activity assay

HEK293 cells were seeded in White 96-well plates (Corning) at 2×10^4 cells/well. After 24 h the cells were transfected with a mixture of DNA and jetPEI (PolyPlus transfection). Total amount of DNA per well was 200 ng. To determine the luciferase activity of plasmids coding tNFAT TFs, cells were transfected with $_{3NFAT}$-P_{min} fLUC reporter plasmid (100 ng/well) or $_{3NFAT}$-CMV fLUC reporter plasmid (100 ng/well) and ABI-tNFAT (2, 5 ng/well) or GAI-tNFAT (2, 5 ng/well) or DmrA-tNFAT (2, 5 ng/well) with corresponding protein partner PYL1-VPR or PYL1-KRAB (5 ng/well), GID1-VPR or GID1-KRAB (5 ng/well), and DmrC-VPR or DmrC-KRAB (5 ng/well). For transfection control, phRL-TK (5 ng DNA/well) was added and an empty vector pcDNA3.1 (Invitrogen) was used to scale DNA up to 200 ng. The next day, the medium was changed and supplemented with 2 mM $CaCl_2$ and 5 μM Ca-ionophore (Sigma Aldrich). For induction of tNFAT TFs heterodimerization ABA (100 μM, Sigma Aldrich), gibberellin (10 μM, Sigma Aldrich) or rapamycin (3 μM, Sigma Aldrich) was added. The concentrations for inducing efficient heterodimerization were determined based on our previous experience.[40,68] Twenty-four hours after heterodimerization induction, cells were harvested and lysed in Passive Lysis Buffer (Promega). The expression of the luciferase reporter genes was analyzed using Dual Glo Luciferase Assay System reagents (Promega) and the Orion luminometer plate reader (Berthold Detection Systems). Relative luciferase activity was calculated by normalizing each sample's firefly luciferase activity with the constitutive Renilla luciferase activity determined within the same sample. Fold activation of reporter expression system was calculated based on RLU values of only pcDNA3-transfected cells.

Cell electroporation

Jurkat cells (3×10^7 cells/mL) were electroporated by Neon electroporation system (Thermo Fisher Scientific), using R buffer in 100 μL electroporation tips (electroporation parameters: 1,600 V voltage, 10 ms pulse width, 3 pulses). The total amount of DNA per electroporation was 10 μg. To determine CD19 CAR-T regulated activity, cells were electroporated with CD19BBz pDNA (5 μg) and ABI-tNFAT (2, 5 μg) or GAI-tNFAT (2, 5 μg) or DmrA-tNFAT (2, 5 μg) with the corresponding protein partner PYL1-VPR or PYL1-KRAB (2, 5 μg), GID1-VPR or GID1-KRAB (2, 5 μg), and DmrC-VPR or DmrC-KRAB (2, 5 μg).

Viral transduction

For retrovirus production, a retrovirus packaging cell line Gryphon Ampho were seeded at 2×10^6 cells/well (6 well). Cells were transfected 24 h later with 3 μg PMX vector, expressing the protein of interest, using jetPEI. At 48 h later, cell supernatant (2 mL) containing retroviral particles was transferred to Retronectin (Takara) coated plates. 1×10^6 T cells/mL were transduced with retroviruses at a MOI 1 for 48 h (ratio of transduction units for CD19BBz: tNFAT TF = 1:1). After that, T cells were subjected to flow cytometry to determine transduction efficiency. Next, cells were expanded at least for 5–7 days in the presence of hIL2 (10 ng/mL). To determine CD19 CAR-T cell activity, T cells were co-cultured with $CD19^+$ target cell line at ratio E:T = 10:1.

For lentivirus production HEK293-T cells were seeded at 4×10^6 cells/plate. Cells were transfected 24 h later with 15 μg LVX vector, expressing protein of interest, 10 μg VSV-G vector, and 5 μg PAX2 vector using jetPEI. Cell supernatant, containing viral particles, was filtrated through a 45-μm filter 48 h later (Sartorius) and afterward placed in ultracentrifuge 25-mL tubes (Beckman Coulter). Tubes were ultracentrifuged at 4°C for 2 h/100,000G (Beckman Coulter). Viral particle pellet was resuspended in PBS. We transduced 1×10^6 T cells with lentiviruses at a MOI of 5–10. Cells were then expanded for $\geq$5–10 days in the presence of hIL2 (50 U/mL). To determine CD19 CAR-T cell activity, T cells were co-cultured with a $CD19^+$ target cell line.

For hCD19-MDA-BR-231-fLUC generation, cells were seeded at 1×10^5 cells/well in a 12-well plate. The next day, they were transduced with lentiviruses in the presence of 8 μg/mL polybrene (Invivogen). Seven days later, hCD19-positive cells were selected by using 0.5 mg/mL of puromycin selection (Invitrogen) for 3 weeks. After

the selection, cells were checked for hCD19 expression, using flow cytometry.

Cytotoxicity determination

To determine cell death several methods were used. LDH cytotoxicity assay (Thermo Fisher Scientific) were carried out according to the manufacturer's protocol. Determination of bioluminescence (BLI) loss was used as a marker for CD19 CAR-T-mediated cell killing of $CD19^+$ BCWM-fLUC cells. After designed period of time, 500 μM D-luciferin (Xenogen) was added to co-cultured cells. BLI was measured using IVIS Lumina Series III (PerkinElmer). Data were analyzed with Living Image 4.5.2 (PerkinElmer). From average radiance values (ARV) the percentage of specific lysis was calculated using formula: % specific lysis = 100 × (spontaneous death ARV–test ARV)/(spontaneous death ARV–maximal killing ARV).

ELISA

Values of human IL2 and IFNγ were determined by using Human IL2 ELISA Kit (Invitrogen) and Human IFNγ (eBioscience) according to the manufacturer's protocol.

Flow cytometry analysis

To determine T cell activation, cells were stained with anti-CD69-FITC antibody (Miltenyi; dilution 1:100), whereas cell staining was performed according to the manufacturer's instructions. For T cell transduction efficiency with CD19 CAR retroviruses, T cells were stained with anti-myc antibody Myc-Tag (9B11) Mouse mAb (Anti Myc) (Cell Signaling; dilution 1:100) and afterward with Alexa Fluor 488 goat anti-mouse IgG (Invitrogen; dilution 1:2,000) secondary antibody. Flow cytometry analysis was performed with flow cytometer CyFlow (Partec). Cells were washed with FACS buffer (PBS, 2% FBS) and resuspended in a 0.1-mL FACS buffer. To determine T cell proliferation status, a CellTrace CFSE Cell Proliferation Kit (Thermo Fisher Scientific) was used according to manufacturer's protocol. An 488-nm diode laser was used. To determine phenotype of the CD19 CAR-T cells, which were daily stimulated with 100 μM ABA without target cell stimulation, the cells were stained with anti-human CD45RA BV421 (Biolegend; dilution 1:50) and with anti-human CD69L AF488 Antibody (Biolegend; dilution 1:50). For $_{fl}$NFAT CAR-T cells, FITC anti-human CD62L (Biolegend; 1:50), Pacific Blue, anti-human CD45RA Antibody (Biolegend; dilution 1:50), CD8-FITC (BW135/80) human, CD4-VioBlue, human (2996, Flow-H2, Miltenyi), MYC-TAG (9B11) MOUSE MAB (Alexa FLU 647) were used.

To check hCD19-positive MDA-MB-BR-231-fLUC cells, cells were stained with hCD19-APC antibody (Miltenyi; dilution 1:50). Data were analyzed with FlowJo software (Tree Star).

Mouse studies

All animal experiments were performed according to the directives of the EU 2010/63 and were approved by the Administration of the Republic of Slovenia for Food Safety, Veterinary Sector and Plant Protection of the Ministry of Agriculture, Forestry and Foods, Republic of Slovenia (Permit Number U34401-28/2019/8). Laboratory animals were housed in IVC cages GM500 (Techniplast), fed standard chow (Mucedola), and tap water was provided ad libitum. The cages were enriched using Nestlets nesting material and mouse houses. Mice were maintained in a 12–12 h dark–light cycle at approximately 40%–60% relative humidity with 22°C of ambient temperature. All animals, used in the study were healthy, accompanied by a health certificate from the animal vendor. Health and microbiological statuses were confirmed by the Federation of European Laboratory Animal Science Associations recommended Mouse Vivum immunocompetent panel (QM Diagnostics).

To test the CD19 CAR-T cell activation cell sensor, female 8- to 10-week-old SCID C.B-17/IcrHsd-Prkdcscid mice (Envigo) were used for xenograft cancer studies. We injected 1×10^6 CD19 CAR-$T_{Akaluc\ sens}$ cells intraperitoneally. The next day, 1×10^6 of Raji cells were again injected intraperitoneally. Three days thereafter, mice underwent live imaging. The mice received 150 mg/kg of body weight of AkaLumine-Hcl (Sigma) intraperitoneally and were *in vivo* imaged with IVIS Lumina Series III (PerkinElmer). Data were analyzed with Living Image 4.5.2 (PerkinElmer).

To determine killing efficiency of CD19 CAR-T cells and ABA AA cells, again male or female SCID 8- to 10-week-old SCID C.B-17/IcrHsd-Prkdcscid mice (Envigo) were used for xenograft cancer studies. Mice were intraperitoneally injected with 1×10^6 of BCWM-fLUC cells. One week later, 5×10^6 of depicted CD19 CAR-T cells were administered intraperitoneally. Mice received daily dose of 100 μL of 100 μM ABA intraperitoneally. The cancer growth and CD19 CAR-T cell therapeutic efficiency was monitored by bioluminiscence detection. Animals were given subcutaneously 150 mg/kg D-luciferin (PerkinElmer). After 10 min, mice were anesthetized by isoflurane inhalation anesthesia and BLI was captured with IVIS Lumina Series III (PerkinElmer). Data were analyzed with Living Image 4.5.2 (PerkinElmer). Blinding of the animal study was conducted as researchers was not aware of the treatment given to each experimental group of the animals.

Statistical analyses

Data are presented as means ± SEM. One-way ANOVA followed by Tukey's multiple comparisons test was used for the statistical comparison of data. Survival curve was analyzed by Mantle-Cox.

DATA AVAILABILITY

Relevant source data are provided with this paper and in supplemental information. All other data are available from the authors of the paper upon request.

SUPPLEMENTAL INFORMATION

Supplemental information can be found online at https://doi.org/10.1016/j.omto.2023.04.005.

ACKNOWLEDGMENTS

We thank Anja Perčič and Klementina Podgoršek for help regarding maintenance of the mouse colony. We are grateful to Mojca Skrbinek and Jure Bohinc for their technical help. This work was supported by grants from the Slovenian Research Agency (P4-0176, J7-4640, Z3-9276, J4-4563).

AUTHOR CONTRIBUTIONS

D.L. and R.J. designed the experiments, with the help of the other authors. D.L., A.G.U, J.P.Ž., V.M., and Š.M. performed the experimental work. D.L. performed animal experiments. V.M. prepared human CD3 cells. R.J. supervised the study. All the authors analyzed and discussed the results. D.L., A.G.U. and R.J. wrote the manuscript. All the authors discussed and commented on the manuscript before submission.

DECLARATION OF INTERESTS

The authors declare no competing financial interest.

REFERENCES

1. Johnson, L.A., and June, C.H. (2017). Driving gene-engineered T cell immunotherapy of cancer. Cell Res. *27*, 38–58.
2. Louis, C.U., Savoldo, B., Dotti, G., Pule, M., Yvon, E., Myers, G.D., Rossig, C., Russell, H.V., Diouf, O., Liu, E., et al. (2011). Antitumor activity and long-term fate of chimeric antigen receptor – positive T cells in patients with neuroblastoma. Mol. Ther. J. Am. Soc. Gene Ther. *118*, 6050–6056.
3. Jensen, M.C., and Riddell, S.R. (2015). Designing chimeric antigen receptors to effectively and safely target tumors. Curr. Opin. Immunol. *33*, 9–15.
4. Fesnak, A.D., June, C.H., and Levine, B.L. (2016). Engineered T cells: the promise and challenges of cancer immunotherapy. Nat. Rev. Cancer *16*, 566–581.
5. Neelapu, S.S., Locke, F.L., Bartlett, N.L., Lekakis, L.J., Miklos, D.B., Jacobson, C.A., Braunschweig, I., Oluwole, O.O., Siddiqi, T., Lin, Y., et al. (2017). Axicabtagene ciloleucel CAR T-cell therapy in refractory large B-cell lymphoma | enhanced reader. N. Engl. J. Med. *377*, 2531–2544.
6. Sterner, R.C., and Sterner, R.M. (2021). CAR-T cell therapy: current limitations and potential strategies. Blood Cancer J. *11*, 69.
7. Tian, Y., Li, Y., Shao, Y., and Zhang, Y. (2020). Gene modification strategies for next-generation CAR T cells against solid cancers. J. Hematol. Oncol. *13*, 16–54.
8. Bonifant, C.L., Jackson, H.J., Brentjens, R.J., and Curran, K.J. (2016). Toxicity and management in CAR T-cell therapy. Mol. Ther. Oncolytics *3*, 16011.
9. Turtle, C.J., Hanafi, L.A., Berger, C., Gooley, T.A., Cherian, S., Hudecek, M., Sommermeyer, D., Melville, K., Pender, B., Budiarto, T.M., et al. (2016). CD19 CAR – T cells of defined CD4 + : CD8 + composition in adult B cell ALL patients. J. Clin. Invest. *126*, 2123–2138.
10. Yeku, O.O., and Brentjens, R.J. (2016). Armored CAR T-cells: utilizing cytokines and pro-inflammatory ligands to enhance CAR T-cell anti-tumour efficacy. Biochem. Soc. Trans. *44*, 412–418.
11. Hawkins, E.R., D'souza, R.R., and Klampatsa, A. (2021). Armored CAR T-cells: the next chapter in T-cell cancer immunotherapy. Biologics. *15*, 95–105.
12. Pietrobon, V., Todd, L.A., Goswami, A., Stefanson, O., Yang, Z., and Marincola, F. (2021). Improving car t-cell persistence. Int. J. Mol. Sci. *22*, 10828.
13. Uchibori, R., Teruya, T., Ido, H., Ohmine, K., Sehara, Y., Urabe, M., Mizukami, H., Mineno, J., and Ozawa, K. (2019). Functional analysis of an inducible promoter driven by activation signals from a chimeric antigen receptor. Mol. Ther. Oncolytics. https://doi.org/10.1016/j.omto.2018.11.003.
14. Zimmermann, K., Kuehle, J., Dragon, A.C., Galla, M., Kloth, C., Rudek, L.S., Sandalcioglu, I.E., Neyazi, B., Moritz, T., Meyer, J., et al. (2020). Design and characterization of an "all-in-one" lentiviral vector system combining constitutive anti-GD2 CAR expression and inducible cytokines. Cancers *12*, 375.
15. Sahillioglu, A.C., Toebes, M., Apriamashvili, G., Gomez, R., and Schumacher, T.N. (2021). Crash-it switch enables reversible and dose-dependent control of tcr and car t-cell function. Cancer Immunol. Res. *9*, 999–1007.
16. Park, S., Pascua, E., Lindquist, K.C., Kimberlin, C., Deng, X., Mak, Y.S.L., Melton, Z., Johnson, T.O., Lin, R., Boldajipour, B., et al. (2021). Direct control of CAR T cells through small molecule-regulated antibodies. Nat. Commun. *12*, 710–713.
17. Rafiq, S., Yeku, O.O., Jackson, H.J., Purdon, T.J., van Leeuwen, D.G., Drakes, D.J., Song, M., Miele, M.M., Li, Z., Wang, P., et al. (2018). Targeted delivery of a PD-1-blocking scFv by CAR-T cells enhances anti-tumor efficacy in vivo. Nat. Biotechnol. *36*, 847–856.
18. Tao, L., Farooq, M.A., Gao, Y., Zhang, L., Niu, C., Ajmal, I., Zhou, Y., He, C., Zhao, G., Yao, J., et al. (2020). CD19-CAR-T cells bearing a KIR/PD-1-Based inhibitory CAR eradicate CD19 + HLA-C1 - malignant B cells while sparing CD19 + HLA-C1 + healthy B cells. Cancers (Basel) *12*, 2612–2617.
19. Foster, A.E., Mahendravada, A., Shinners, N.P., Chang, W.-C., Crisostomo, J., Lu, A., Khalil, M., Morschl, E., Shaw, J.L., Saha, S., et al. (2017). Regulated expansion and survival of chimeric antigen receptor-modified T cells using small molecule-dependent inducible MyD88/CD40. Mol. Ther. *25*, 2176–2188.
20. Wu, C.-Y., Roybal, K.T., Puchner, E.M., Onuffer, J., and Lim, W.A. (2015). Remote control of therapeutic T cells through a small molecule-gated chimeric receptor. Science *350*, aab4077.
21. Morsut, L., Roybal, K.T., Xiong, X., Gordley, R.M., Coyle, S.M., Thomson, M., and Lim, W.A. (2016). Engineering customized cell sensing and response behaviors using synthetic notch receptors. Cell *164*, 780–791.
22. Li, H.-S., Israni, D.V., Gagnon, K.A., Gan, K.A., Raymond, M.H., Sander, J.D., Roybal, K.T., Joung, J.K., Wong, W.W., and Khalil, A.S. (2022). Multidimensional control of therapeutic human cell function with synthetic gene circuits. Science *378*, 1227–1234.
23. Głowacki, P., and Rieske, P. (2022). Application and design of switches used in CAR. Cells *11*, 1910.
24. Cho, J.H., Okuma, A., Sofjan, K., Lee, S., Collins, J.J., and Wong, W.W. (2021). Engineering advanced logic and distributed computing in human CAR immune cells. Nat. Commun. *12*, 792.
25. Macian, F. (2005). NFAT proteins: key regulators of T-cell development and function. Nat. Rev. Immunol. *5*, 472–484.
26. Huang, Z., Wu, Y., Allen, M.E., Pan, Y., Kyriakakis, P., Lu, S., Chang, Y.J., Wang, X., Chien, S., and Wang, Y. (2020). Engineering light-controllable CAR T cells for cancer immunotherapy. Sci. Adv. *6*, eaay9209.
27. Lee, S.M., Kang, C.H., Choi, S.U., Kim, Y., Hwang, J.Y., Jeong, H.G., and Park, C.H. (2020). A chemical switch system to modulate chimeric antigen receptor T cell activity through proteolysis-targeting chimaera technology. ACS Synth. Biol. *9*, 987–992.
28. Zajc, C.U., Dobersberger, M., Schaffner, I., Mlynek, G., Pühringer, D., Salzer, B., Djinović-Carugo, K., Steinberger, P., de Sousa Linhares, A., Yang, N.J., et al. (2020). A conformation-specific ON-switch for controlling CAR T cells with an orally available drug. Proc. Natl. Acad. Sci. USA *117*, 14926–14935.
29. Milone, M.C., Xu, J., Chen, S.J., Collins, M.A., Zhou, J., Powell, D.J., and Melenhorst, J.J. (2021). Engineering-enhanced CAR T cells for improved cancer therapy. Nat. Cancer *2*, 780–793.
30. Salzer, B., Schueller, C.M., Zajc, C.U., Peters, T., Schoeber, M.A., Kovacic, B., Buri, M.C., Lobner, E., Dushek, O., Huppa, J.B., et al. (2020). Engineering AvidCARs for combinatorial antigen recognition and reversible control of CAR function. Nat. Commun. *11*, 4166.
31. Leung, W.H., Gay, J., Martin, U., Garrett, T.E., Horton, H.M., Certo, M.T., Blazar, B.R., Morgan, R.A., Gregory, P.D., Jarjour, J., and Astrakhan, A. (2019). Sensitive and adaptable pharmacological control of CAR T cells through extracellular receptor dimerization. JCI Insight *5*, e124430.
32. Chaoul, N., Fayolle, C., Desrues, B., Oberkampf, M., Tang, A., Ladant, D., and Leclerc, C. (2015). Microenvironment and immunology rapamycin impairs antitumor CD8 þ T-cell responses and vaccine-induced tumor eradication. Cancer Res. *75*, 3279–3291.
33. Caulier, B., Enserink, J.M., and Wälchli, S. (2021). Pharmacologic control of CAR T cells. Int. J. Mol. Sci. *22*, 4320.

34. Labanieh, L., Majzner, R.G., Klysz, D., Sotillo, E., Fisher, C.J., Vilches-Moure, J.G., Pacheco, K.Z.B., Malipatlolla, M., Xu, P., Hui, J.H., et al. (2022). Enhanced safety and efficacy of protease-regulated CAR-T cell receptors. Cell *185*, 1745–1763.e22.

35. Li, H.S., Wong, N.M., Tague, E., Ngo, J.T., Khalil, A.S., and Wong, W.W. (2022). High-performance multiplex drug-gated CAR circuits. Cancer Cell *40*, 1294–1305.e4.

36. Kaminuma, O., Kitamura, F., Kitamura, N., Hiroi, T., Miyoshi, H., Miyawaki, A., and Miyatake, S. (2008). Differential contribution of NFATc2 and NFATc1 to TNF- gene expression in T cells. J. Immunol. *180*, 319–326.

37. Hogan, P.G. (2017). Calcium–NFAT transcriptional signalling in T cell activation and T cell exhaustion. Cell Calcium *63*, 66–69.

38. Ross, S.H., and Cantrell, D.A. (2018). Signaling and function of interleukin-2 in T lymphocytes. Annu. Rev. Immunol. *36*, 411–433.

39. Meško, M., Lebar, T., Dekleva, P., Jerala, R., and Benčina, M. (2020). Engineering and rewiring of a calcium-dependent signaling pathway. ACS Synth. Biol. *9*, 2055–2065.

40. Fink, T., Lonzarić, J., Praznik, A., Plaper, T., Merljak, E., Leben, K., Jerala, N., Lebar, T., Strmšek, Ž., Lapenta, F., et al. (2019). Design of fast proteolysis-based signaling and logic circuits in mammalian cells. Nat. Chem. Biol. *15*, 115–122.

41. Chavez, A., Scheiman, J., Vora, S., Pruitt, B.W., Tuttle, M., P R Iyer, E., Lin, S., Kiani, S., Guzman, C.D., Wiegand, D.J., et al. (2015). Highly efficient Cas9-mediated transcriptional programming. Nat. Methods *12*, 326–328.

42. Maeder, M.L., Linder, S.J., Cascio, V.M., Fu, Y., Ho, Q.H., and Joung, J.K. (2013). CRISPR RNA-guided activation of endogenous human genes. Nat. Methods *10*, 977–979.

43. Woerly, G., Brooks, N., and Ryffel, B. (1996). Effect of rapamycin on the expression of the IL-2 receptor (CD25). Clin. Exp. Immunol. *103*, 322–327.

44. Crabtree, G.R., and Olson, E.N. (2002). NFAT signaling: choreographing the social lives of cells. Cell *109*, S67–S79.

45. Badran, B.M., Wolinsky, S.M., Burny, A., and Willard-Gallo, K.E. (2002). Identification of three NFAT binding motifs in the 5-upstream region of the human CD3gamma gene that differentially bind NFATc1, NFATc2, and NF-kappa B p50. J. Biol. Chem. *277*, 47136–47148.

46. Wu, H.D., Kikuchi, M., Dagliyan, O., Aragaki, A.K., Nakamura, H., Dokholyan, N.V., Umehara, T., and Inoue, T. (2020). Rational design and implementation of a chemically inducible hetero-trimerization system. Nat. Methods *17*, 928–936.

47. Huang, R., Li, X., He, Y., Zhu, W., Gao, L., Liu, Y., Gao, L., Wen, Q., Zhong, J.F., Zhang, C., and Zhang, X. (2020). Recent advances in CAR-T cell engineering. J. Hematol. Oncol. *13*, 19–84.

48. Ditzel Santos, D., Ho, A.W., Tournilhac, O., Hatjiharissi, E., Leleu, X., Xu, L., Tassone, P., Neri, P., Hunter, Z.R., Chemaly, M.A.Z., et al. (2007). Establishment of BCWM.1 cell line for Waldenström's macroglobulinemia with productive in vivo engraftment in SCID-hu mice. Exp. Hematol. *35*, 1366–1375.

49. Tantalo, D.G., Oliver, A.J., Von Scheidt, B., Harrison, A.J., Mueller, S.N., Kershaw, M.H., and Slaney, C.Y. (2021). Understanding T cell phenotype for the design of effective chimeric antigen receptor T cell therapies. J. Immunother. Cancer *9*, e002555.

50. Singh, A.K., and McGuirk, J.P. (2020). CAR T cells: continuation in a revolution of immunotherapy. Lancet Oncol. *21*, e168–e178.

51. Donnadieu, E., Dupré, L., Pinho, L.G., and Cotta-de-Almeida, V. (2020). Surmounting the obstacles that impede effective CAR T cell trafficking to solid tumors. J. Leukoc. Biol. *108*, 1067–1079.

52. Iwano, S., Sugiyama, M., Hama, H., Watakabe, A., Hasegawa, N., Kuchimaru, T., Tanaka, K.Z., Takahashi, M., Ishida, Y., Hata, J., et al. (2018). Single-cell bioluminescence imaging of deep tissue in freely moving animals. Science *359*, 935–939.

53. Bozec, D., Sattiraju, A., Bouras, A., Jesu Raj, J.G., Rivera, D., Huang, Y., Junqueira Alves, C., Tejero, R., Tsankova, N.M., Zou, H., et al. (2020). Akaluc bioluminescence offers superior sensitivity to track in vivo glioma expansion. Neurooncol. Adv. *2*, vdaa134.

54. Macián, F., López-Rodríguez, C., and Rao, A. (2001). Partners in transcription: NFAT and AP-1. Oncogene *20*, 2476–2489.

55. Klein-Hessling, S., Muhammad, K., Klein, M., Pusch, T., Rudolf, R., Flöter, J., Qureischi, M., Beilhack, A., Vaeth, M., Kummerow, C., et al. (2017). NFATc1 controls the cytotoxicity of CD8+ T cells. Nat. Commun. *8*, 511.

56. Chuvpilo, S., Jankevics, E., Tyrsin, D., Akimzhanov, A., Moroz, D., Jha, M.K., Schulze-Luehrmann, J., Santner-Nanan, B., Feoktistova, E., König, T., et al. (2002). Autoregulation of NFATc1/A expression facilitates effector T cells to escape from rapid apoptosis. Immunity *16*, 881–895.

57. Ogishi, M., and Yotsuyanagi, H. (2019). Quantitative prediction of the landscape of T cell epitope immunogenicity in sequence space. Front. Immunol. *10*, 827.

58. Moghanloo, E., Mollanoori, H., Talebi, M., Pashangzadeh, S., Faraji, F., Hadjilooei, F., and Mahmoodzadeh, H. (2021). Remote controlling of CAR-T cells and toxicity management: molecular switches and next generation CARs. Transl. Oncol. *14*, 101070.

59. Jin, C., Ma, J., Ramachandran, M., Yu, D., and Essand, M. (2022). CAR T cells expressing a bacterial virulence factor trigger potent bystander antitumour responses in solid cancers. Nat. Biomed. Eng. *6*, 830–841.

60. Magnone, M., Sturla, L., Guida, L., Spinelli, S., Begani, G., Bruzzone, S., Fresia, C., and Zocchi, E. (2020). Abscisic acid: a conserved hormone in plants and humans and a promising aid to combat prediabetes and the metabolic syndrome. Nutrients. https://doi.org/10.3390/nu12061724.

61. Liang, F.-S., Ho, W.Q., and Crabtree, G.R. (2008). Engineering the ABA plant stress pathway for regulation of induced proximity. Sci. Signal. https://doi.org/10.1126/scisignal.2001449.

62. Guri, A.J., Hontecillas, R., and Bassaganya-Riera, J. (2010). Abscisic acid synergizes with rosiglitazone to improve glucose tolerance, down-modulate macrophage accumulation in adipose tissue: possible action of the cAMP/PKA/PPAR γ axis. Clin. Nutr. *29*, 646–653.

63. Guri, A.J., Misyak, S.A., Hontecillas, R., Hasty, A., Liu, D., Si, H., and Bassaganya-Riera, J. (2010). Abscisic acid ameliorates atherosclerosis by suppressing macrophage and CD4 + T cell recruitment into the aortic wall. J. Nutr. Biochem. *21*, 1178–1185.

64. Martinez, G.J., Pereira, R.M., Äijö, T., Kim, E.Y., Marangoni, F., Pipkin, M.E., Togher, S., Heissmeyer, V., Zhang, Y.C., Crotty, S., et al. (2015). The transcription factor NFAT promotes exhaustion of activated CD8+T cells. Immunity *42*, 265–278.

65. Bengsch, B., and Wherry, E.J. (2015). The importance of cooperation: partnerless NFAT induces T cell exhaustion. Immunity *42*, 203–205.

66. Cazaux, M., Grandjean, C.L., Lemaître, F., Garcia, Z., Beck, R.J., Milo, I., Postat, J., Beltman, J.B., Cheadle, E.J., and Bousso, P. (2019). Single-cell imaging of CAR T cell activity in vivo reveals extensive functional and anatomical heterogeneity. J. Exp. Med. *216*, 1038–1049.

67. Mues, M., Bartholomäus, I., Thestrup, T., Griesbeck, O., Wekerle, H., Kawakami, N., and Krishnamoorthy, G. (2013). Real-time in vivo analysis of T cell activation in the central nervous system using a genetically encoded calcium indicator. Nat. Med. *19*, 778–783.

68. Lebar, T., Lainšček, D., Merljak, E., Aupič, J., and Jerala, R. (2020). A tunable orthogonal coiled-coil interaction toolbox for engineering mammalian cells. Nat. Chem. Biol. *16*, 513–519.

OMTO, Volume *29*

Supplemental information

Regulation of CD19 CAR-T cell activation based on an engineered downstream transcription factor

Duško Lainšček, Anja Golob-Urbanc, Veronika Mikolič, Jelica Pantović-Žalig, Špela Malenšek, and Roman Jerala

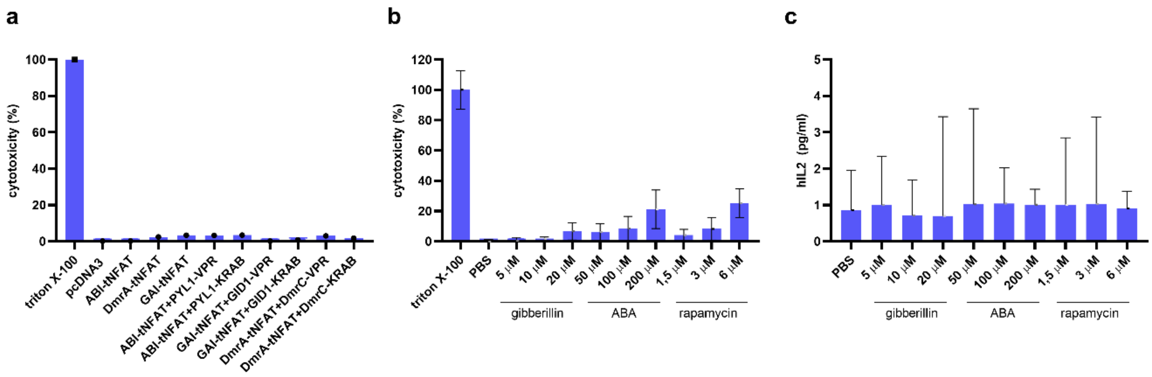

Figure S1: tNFAT transcription factors influence cell viability and activation. Jurkat cells ($3x10^7$ cells/ml) were Neon electroporated with listed plasmid DNA for expression of tNFAT transcription factors. 48 hours later LDH release was determined in cell media and % of cytotoxicity was calculated (a). Jurkat cells ($1x10^6$ cells/ml) were treated with various concentrations of heterodimerization inductors and 48 hours later cytotoxicity was determined based on LDH release (b) and IL2 secretion was quantified using ELISA (c). Data present three individual separate experiments (n=3).

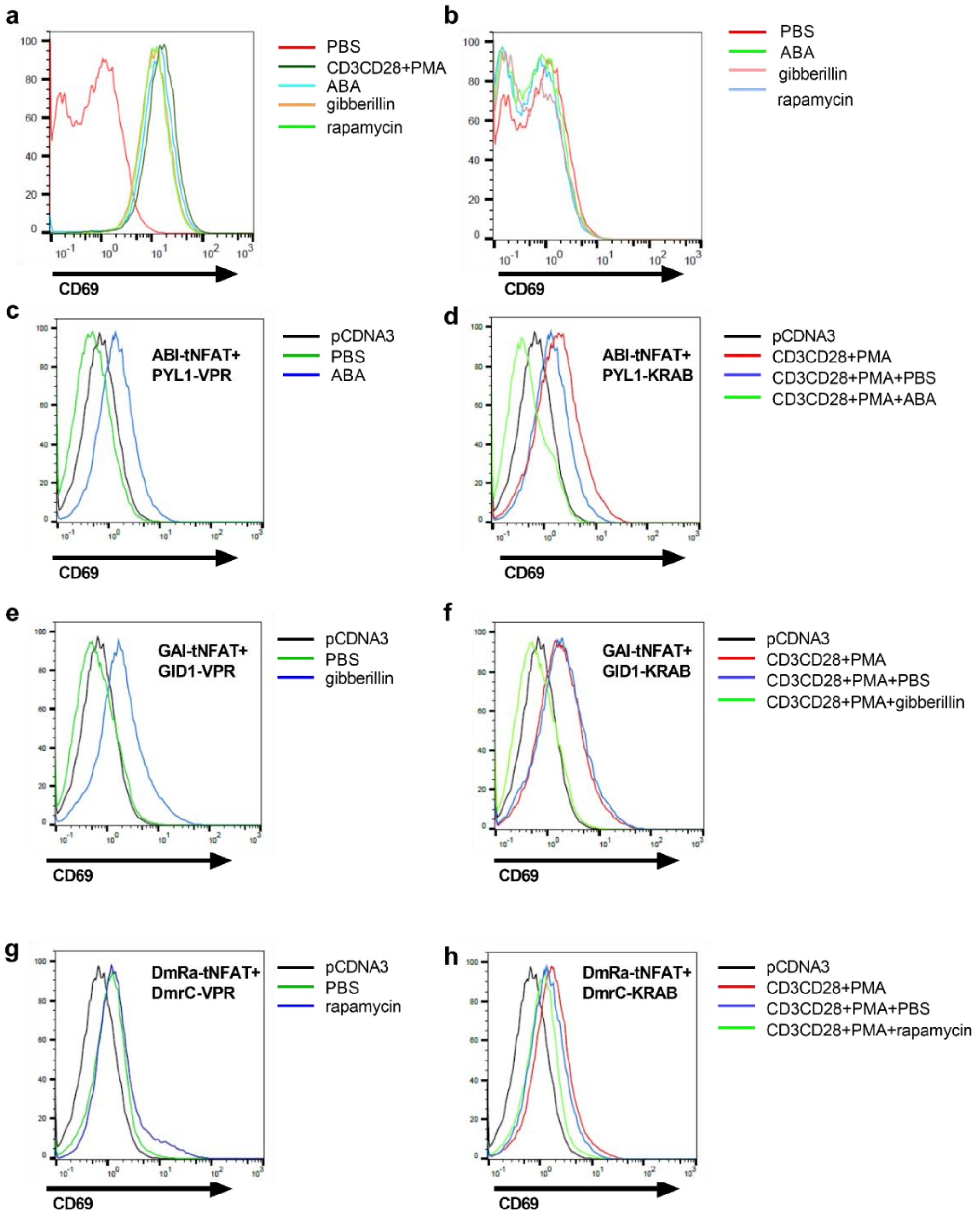

Figure S2: Regulation of T cell activation based on tNFAT transcription factors. Jurkat cells ($3x10^7$ cells/ml) were Neon electroporated with depicted plasmid DNA for expression of tNFAT transcription factors and stained against CD69 to determine cell activation. Jurkat cells were electroporated with empty pcDNA3 plasmid and 24 hours later treated with CD3CD28 Dynabeads (beads:cells=1:1)+PMA (50ng/ml). The next day cells were stimulated with HD inductors (ABA-100 µM; Gibberillin-10 µM or rapamycin- 3 µM). 24 hours later CD69 cell surface expression was determined (a). pcDNA3 electroporated Jurkat cells were stimulated with HD inductors and CD69 was measured (b). tNFAT transcription factors were electroporated into Jurkat cells and cell activation was determined 48 hours later after the

induction of heterodimerization with the addition of ABA (100 μM) (c-d), gibberellin (10 μM) (e-f) or rapamycin (3 μM) (g-h).

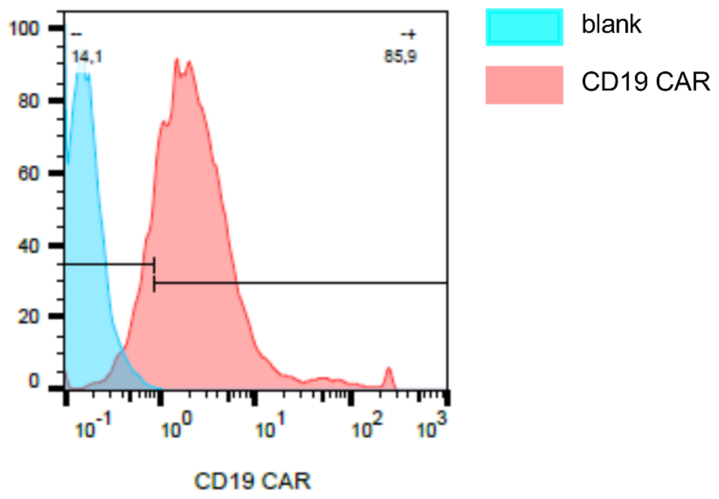

Figure S3: T cell transduction efficiency. To obtain CD19 CAR-T cells, T cells were transduced using retronectin-mediated retroviruses (CD19 CAR) or empty vector (blank). 5 days after the transduction, T cells were stained against the myc tag, expressed on the N-terminus of the CD19 CAR construct, and subjected to flow cytometry.

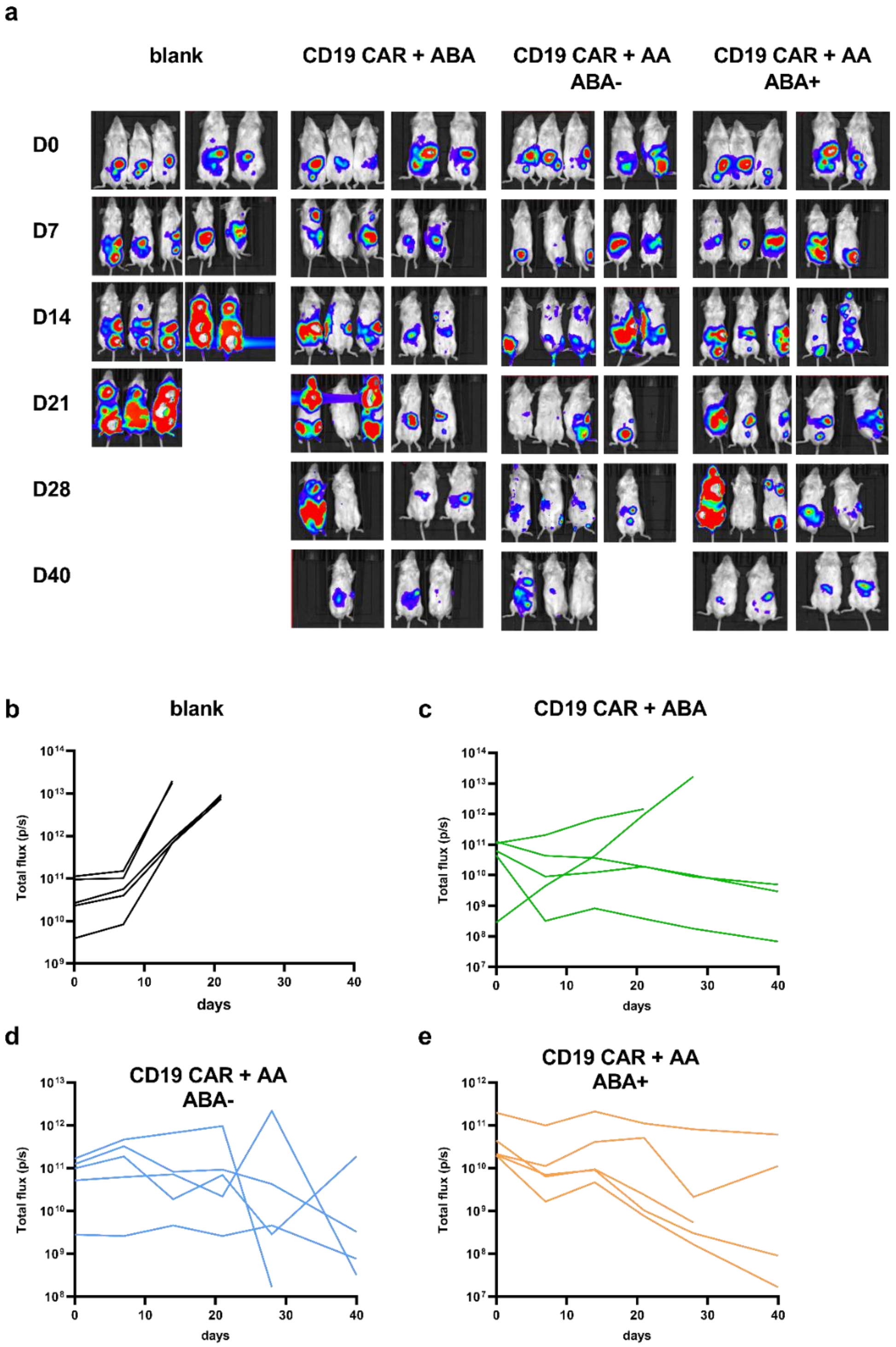

Figure S4: CD19 CAR T cell therapeutic efficiency *in vivo*. A hCD19+ BCWM-fLUC xenograft mouse cancer model was developed (10^6 cells/mouse). CD19 CAR T cells or ABA AA CD19 CAR T cells ($5x10^6$ cells/mouse) were administered. Daily ABA (100 μM) was injected into mice. Mice were subjected to BLI imaging weekly (a). Total flux measurement for cancer cell proliferation in vivo. Each line presents a single animal (b-e).

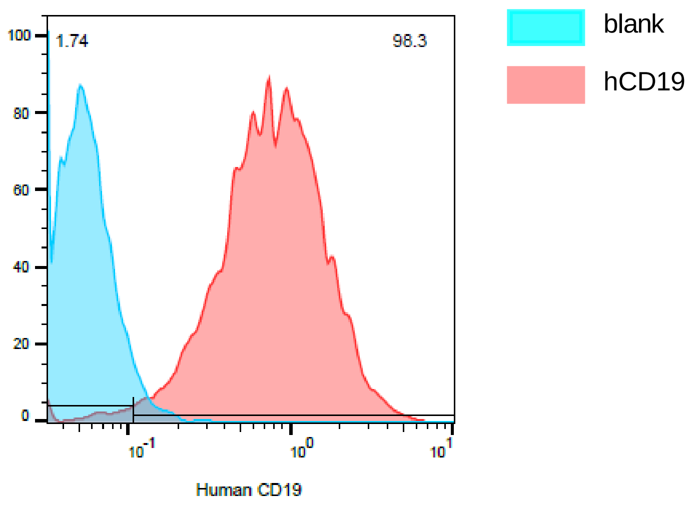

Figure S5: hCD19 MDA-MB-BR-231-fLUC cell generation. To obtain hCD19 MDA-MB-BR-231-fLUC cells, cells were transduced using lentiviruses (hCD19) or empty vector (blank). Three weeks after the puromycin selection, cells were subjected to flow cytometry to determine stable hCD19 integration.

Table S1: Statistical analysis for Total flux analysis (related to Fig 4h)

Tukey's multiple comparisons tests	Mean Diff.	Significant	Summary	P Value
blank vs. CD19 CAR+ ABA	3301934969444	No	ns	0,1066
blank vs. CD19 CAR+ AA (ABA-)	3923746600000	Yes	*	0,0441
blank vs. CD19 CAR+ AA (ABA+)	4095742344444	Yes	*	0,0341
CD19 CAR+ ABA vs. CD19 CAR+ AA (ABA-)	621811630556	No	ns	0,9552
CD19 CAR+ ABA vs. CD19 CAR+ AA (ABA+)	793807375000	No	ns	0,9129
CD19 CAR+ AA (ABA-) vs. CD19 CAR+ AA (ABA+)	171995744444	No	ns	0,9989

Ordinary one-way ANOVA was carried out with Tukey's multiple comparison test.

Table S2: Statistical analysis for Survival analysis (related to Fig 4i)

Log-rank (Mantel-Cox) test	Significant	Summary	P Value
blank vs. CD19 CAR+ ABA	Yes	**	0,0067
blank vs. CD19 CAR+ AA (ABA-)	Yes	*	0,0323
blank vs. CD19 CAR+ AA (ABA+)	Yes	**	0,0029
CD19 CAR+ ABA vs. CD19 CAR+ AA (ABA-)	No	ns	0,9065
CD19 CAR+ ABA vs. CD19 CAR+ AA (ABA+)	No	ns	0,5203
CD19 CAR+ AA (ABA-) vs. CD19 CAR+ AA (ABA+)	No	ns	0,5203

A comparison of survival curves was performed with Long-rank (Mantel-Cox) test.
